# Crystal structure of epidermal growth factor domain-specific *O*-linked *N*-acetylglucosamine transferase reveals a conserved N–R–R constellation for uridine diphosphate recognition in the GT61 family

**DOI:** 10.1093/pnasnexus/pgag115

**Published:** 2026-04-15

**Authors:** Yuko Tashima, Masamichi Nagae, Jiaoyang Jiang, Tetsuya Okajima

**Affiliations:** Department of Molecular Biochemistry, Nagoya University Graduate School of Medicine, 65 Tsurumai-Cho, Showa-Ku, Nagoya, Aichi 466-8550, Japan; Institute for Glyco-Core Research (iGCORE), Nagoya University, Furo-Cho, Chikusa-Ku, Nagoya, Aichi 464-8601, Japan; Department of Molecular Immunology, Research Institute for Microbial Diseases, The University of Osaka, Suita, Osaka 565-0871, Japan; Laboratory of Molecular Immunology, Immunology Frontier Research Center, The University of Osaka, Suita, Osaka 565-0871, Japan; Pharmaceutical Sciences Division, School of Pharmacy, University of Wisconsin—Madison, Madison, WI 53705, USA; Department of Molecular Biochemistry, Nagoya University Graduate School of Medicine, 65 Tsurumai-Cho, Showa-Ku, Nagoya, Aichi 466-8550, Japan; Institute for Glyco-Core Research (iGCORE), Nagoya University, Furo-Cho, Chikusa-Ku, Nagoya, Aichi 464-8601, Japan

**Keywords:** EOGT, *O*-GlcNAc, UDP, glycosyltransferase family 61, Adams–Oliver syndrome

## Abstract

Epidermal growth factor (EGF) domain-specific *O*-linked *N*-acetylglucosamine transferase (EOGT), a glycosyltransferase (GT) 61 family member, catalyzes *O*-*N*-acetylglucosamine (*O*-GlcNAc) transfer from uridine diphosphate (UDP)–GlcNAc to serine or threonine residues within EGF domains in the endoplasmic reticulum. In this study, we determined the crystal structure of the EOGT–UDP complex and identified the critical residues mediating their interactions, which were validated via site-directed mutagenesis and enzyme activity assays. These residues were conserved in EOGT orthologs across metazoans, and UDP binding occurred independently of divalent metal ions and the canonical Asp–X–Asp motif. Although EOGT catalyzes *O*-GlcNAcylation, similar to *O*-GlcNAc transferase (OGT), it shares little sequence similarity with OGT and belongs to a distinct GT family. Instead, EOGT is more closely related to protein *O*-linked-mannose β1,4-*N*-acetylglucosaminyltransferase 2 (POMGNT2). Structural comparison with POMGNT2 revealed a conserved triad of one asparagine and two arginine residues, the N–R–R constellation. These elements were conserved across metazoans and green plants (Viridiplantae), suggesting a unifying mechanism of UDP recognition and providing a framework to interpret disease-associated *EOGT* mutations and assess the evolution of catalytically active GT61 family enzymes.

Significance Statement Epidermal growth factor (EGF) domain-specific *O*-linked *N*-acetylglucosamine transferase (EOGT) is an enzyme that attaches a specific sugar (*O*-*N*-acetylglucosamine, *O*-GlcNAc) to EGF domains of proteins, and its mutations cause congenital disorders such as Adams–Oliver syndrome. However, the molecular basis by which these mutations impair EOGT function has remained unclear. Here, we determine the crystal structure of EOGT bound to uridine diphosphate (UDP) and provide mechanistic insights into how disease-associated mutations disrupt its enzymatic activity. We identify a conserved cluster of three amino acids (Asn–Arg–Arg) that mediates UDP recognition. This structural feature is also found in POMGNT2, a related glycosyltransferase in the same enzyme family, and is conserved across animals and green plants. These findings provide insights into the evolution and catalytic features of this enzyme family.

## Introduction

*O*-GlcNAcylation, the addition of β-*N*-acetylglucosamine to serine or threonine residues, is a dynamic and abundant posttranslational modification in mammalian cells. Intracellular proteins are modified by *O*-*N*-acetylglucosamine (*O*-GlcNAc) transferase (OGT) (1, 2), whereas subsets of extracellular and secreted proteins are modified by epidermal growth factor (EGF) domain-specific *O*-linked GlcNAc transferase (EOGT) (3–5). The discovery of EOGT revealed a previously unrecognized glycosylation pathway for *O*-GlcNAcylation, distinct from the intracellular modification catalyzed by OGT (6). Although both OGT and EOGT transfer *O*-GlcNAc, their amino acid sequence and predicted domain structure of EOGT differ substantially from OGT. Indeed, unlike OGT (7), the crystal structure of EOGT has not yet been reported.

EOGT selectively modifies EGF-like domains harboring a defined consensus motif, C^5^–X–X–X–X–T/S–G–X–X–C^6^. To date, relatively small subsets of proteins, including Notch receptors, have been reported to serve as EOGT substrates (8). Impaired Notch signaling is linked to the Adams–Oliver syndrome (AOS), a rare congenital disorder characterized by scalp aplasia and limb defects (4, 9), with several *EOGT* mutations identified in affected patients (10). Impaired *O*-GlcNAc transferase activity has been reported for AOS-related *EOGT* mutations, including those decreasing uridine diphosphate (UDP)–GlcNAc binding (11). The biological significance of EOGT during vascular and intestinal development has been analyzed using EOGT-knockout mice (12–14).

To date, most biochemical studies in mammals have focused on mouse EOGT, a 527-amino-acid protein characterized by conserved features essential for its function, including a C-terminal His–Asp–Glu–Leu sequence required for endoplasmic reticulum (ER) retention and two *N*-glycosylation sites (N263 and N354) modified with oligomannose-type glycans in HEK293T cells (15). These *N*-glycosylation sites are required for EOGT maturation and peripheral ER localization but not for enzyme activity (15). These features are well conserved between mouse and human EOGT, with ∼90% sequence identity. Immunostaining combined with confocal microscopy has confirmed the localization of EOGT to the ER (3, 15).

Glycosyltransferases (GTs) are classified into distinct structural folds, primarily GT-A and GT-B, which differ in both architecture and catalytic mechanism. GT-A-fold enzymes typically contain a metal-coordinating DxD motif involved in donor substrate binding, whereas GT-B-fold enzymes generally lack such motifs and adopt a bilobal architecture for substrate recognition. Furthermore, GTs are classified into enzyme families based on amino acid sequence similarity, as categorized in the Carbohydrate-Active Enzymes (CAZy) database (16). EOGT belongs to the GT61 family that is predicted to adopt a GT-B fold. However, a DxD-like sequence has been previously noted in EOGT, and alanine substitution of the DYD_295-297_ sequence resulted in decreased *O*-GlcNAcylation of the NOTCH extracellular fragment (17). Therefore, determining the three-dimensional structure of EOGT is essential for clarifying the structural context and potential functional significance of the DYD_295-297_ sequence.

In mammals, protein *O*-linked-mannose β1,4-*N*-acetylglucosaminyltransferase 2 (POMGNT2) is another GT61 family enzyme that catalyzes the GlcNAc modification of *O*-mannosylated dystroglycan in the ER (18, 19). Both enzymes share the common sugar donor substrate of UDP–GlcNAc (16), suggesting that they may represent common structural features in donor substrate recognition. Furthermore, recent molecular docking analyses have predicted similar UDP–sugar donor-binding modes among plant GT61 enzymes (20, 21). Therefore, a precise understanding of the structure of EOGT and its substrate-binding mode may provide a framework for interpreting the evolution of GT61 family enzymes.

Here, we present the first crystal structure of mouse EOGT bound to UDP and identify a conserved triad, which we term the “N–R–R constellation,” required for UDP recognition. These elements are evolutionarily conserved across metazoans and green plants, providing insight into GT61 enzyme evolution and the mechanistic implications of disease-associated *EOGT* mutations.

## Results

### Overall structure of the EOGT–UDP complex

**Figure 1:**
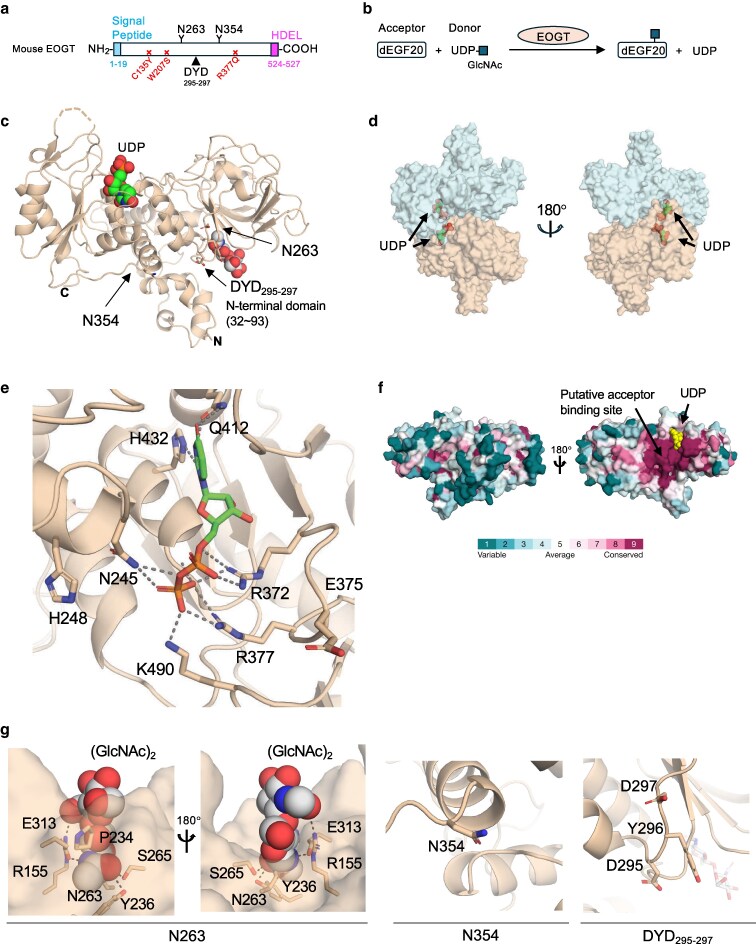
Overall structure and key structural features of the EOGT–UDP complex. a) Schematic representation of mouse EOGT used in this study. EOGT contains a signal sequence, two *N*-glycosylation sites (N263 and N354) indicated by “Y,” an Asp–Tyr–Asp (DYD) sequence (residues 295–297; DYD_295-297_, arrow), and a His–Asp–Glu–Leu (HDEL) sequence that serves as an ER retention signal. Amino acid residues mutated in the AOS (C135Y, W207S, and R377Q) are conserved in mouse and indicated with an “x”. b) EOGT enzymatic reaction using UDP–GlcNAc and *Drosophila* EGF 20 (dEGF20) as substrates. c) Ribbon diagram of the EOGT–UDP complex based on the crystal structure. EOGT adopts a bi-lobe structure, resembling a hand shape. Detailed information on data collection and refinement statistics of the crystal is presented in Table 1. C, C-terminus; N, N-terminus. Arrows indicate the *N*-glycosylation sites (N263 and N354) and DYD_295-297_ sequence. d) Transparent surface representation of the symmetrical dimer of EOGT with UDP detected in this study. e) Close-up view of the UDP-binding site in EOGT. The ribbon diagram illustrates the interaction between EOGT and UDP, with key amino acid residues near the UDP-binding site highlighted in bold. Gray dotted line, hydrogen bond. f) Surface mapping of conserved amino acids. Evolutionary conservation scores were calculated using the ConSurf SERVER (https://colab.research.google.com/drive/1PhDXX7k12oUsV6T_xkXC3Rm9R99e7tHz) with the default setting. Arrow indicates the putative acceptor-binding site. UDP is highlighted in yellow. g) Enlarged view of the two *N*-glycosylation sites (N263 and N354) and DYD_295-297_ sequence. Amino acid residues interacting with the chitobiose moiety of *N*-glycan at N263 are shown. Black dotted line, hydrogen bond.

To facilitate the structural and biochemical studies of EOGT, we established a human embryonic kidney (HEK)-293T cell line stably expressing FLAG-tagged mouse EOGT (Fig. 1a). The protein was purified from the lysate using an anti-FLAG-agarose gel, eluted with FLAG peptides, and treated with HRV 3C protease to remove the FLAG tag. The resulting EOGT was further purified via size-exclusion chromatography. EOGT contained a signal sequence at the N-terminus, followed by two catalytic lobes and a C-terminal His–Asp–Glu–Leu sequence required for ER retention.

To obtain the structure of EOGT, the purified EOGT enzyme was mixed with UDP instead of UDP–GlcNAc to arrest the reaction (Fig. 1b) and subjected to co-crystallization. Notably, we successfully determined the crystal structure of the EOGT–UDP complex at 1.8 Å resolution (Fig. 1c; Table 1). In the crystal packing, this complex was observed as a symmetric dimer (Fig. 1d), in which the putative catalytic sites faced each other. The overall architecture of EOGT showed a bilobed structure with an additional N-terminal domain (P32–G93) that resembled an open glove. This is a typical GT-B fold containing two Rossmann-like domains (22). UDP was located in the cleft between the two lobes. The EOGT–UDP interaction was mediated by multiple residues, including N245, H248, R372, R377, Q412, and H432 (Figs. 1e, f and S1).

**Table 1:** Data collection and refinement statistics.

	EOGT–UDP complex
Resolution (Å)	49.08–1.8 (1.864–1.8)
Space group	*P* 2_1_ 2 2_1_
Unit cell	*a* = 54.9, *b* = 58.6, *c* = 179.4 Å
Total reflections	109,240 (10,711)
Unique reflections	54,653 (5,364)
Multiplicity	2.0 (2.0)
Completeness (%)	99.92 (99.67)
Mean *I*/*σ*(*I*)	15.38 (2.93)
Wilson *B*-factor (Å^2^)	23.6
*R*_merge_ (%)	2.752 (25.71)
*R*_meas_ (%)	3.892 (36.36)
*R*_pim_ (%)	2.752 (25.71)
CC1/2	0.999 (0.869)
CC*	1 (0.964)
Reflections used in refinement	54,641 (5,361)
Reflections used for *R*_free_	2,732 (269)
*R*_work_ (%)	17.20 (21.44)
*R*_free_ (%)	20.14 (27.92)
CC (work)	0.964 (0.911)
CC (free)	0.959 (0.841)
Number of nonhydrogen Atoms	4,326
Macromolecules	4,002
Ligands	32
Solvents	292
Protein residues	480
RMS (bond lengths, Å)	0.01
RMS (bond angles, °)	1.08
Ramachandran favored (%)	97.48
Ramachandran allowed (%)	2.52
Ramachandran outliers (%)	0
Rotamer outliers (%)	0
Clashscore	3.3
Average *B* factor (Å^2^)	28.79
Macromolecules	28.33
Ligands	35.25
Solvents	35.24

Statistics for the highest-resolution shell are shown in parentheses. EOGT, epidermal growth factor domain-specific *O*-linked *N*-acetylglucosamine transferase; UDP, uridine diphosphate; CC, correlation coefficient.

A previous study indicated that EOGT is modified with *N*-glycans at N263 and N354, which are required for EOGT maturation and peripheral ER localization, but not for enzyme activity (15). Consistently, N263 and N354 were located on the opposite side of the predicted active site (Fig. 1g). Importantly, clear electron density revealed the presence of a chitobiose moiety (GlcNAc)_2_ at N263 and interacting amino acid residues R155, P234, Y236, S265, and E313. The chitobiose unit formed hydrogen bonds between the first GlcNAc residue and the side chains of Y236, S265, and E313, whereas the second GlcNAc interacted with R155. In contrast, no clear electron density corresponding to a GlcNAc moiety was observed at N354. Additionally, the GlcNAc engaged in van der Waals interactions with P234. Our structure demonstrated that the DYD_295-297_ sequence did not participate in metal coordination or direct UDP binding (Fig. 1G), confirming that EOGT lacks a canonical DxD motif in functional terms. Overall, our analysis revealed the first detailed architecture of the EOGT–UDP complex, providing a basis for further functional studies.

### Evolutionary conservation of the structural features of EOGT

Since EOGT enzymatic activity and function have been demonstrated in *Drosophila* (3, 17), we examined whether EOGT orthologs are conserved in evolutionarily ancient species beyond Arthropoda. Database analysis revealed that EOGT was conserved in Porifera and Cnidaria, two of the earliest-diverging metazoan lineages (Fig. S1). Importantly, six amino acid residues mediating UDP interactions were invariant, supporting conserved GT activity. Furthermore, the *N*-glycosylation site at N263 was also invariant, and R155, P234, and S265, which interacted with the chitobiose moiety of *N*-glycan, were highly preserved, suggesting the important roles of *N*-glycan in EOGT functions.

### Structural and functional analysis of the UDP-binding site of EOGT

**Figure 2:**
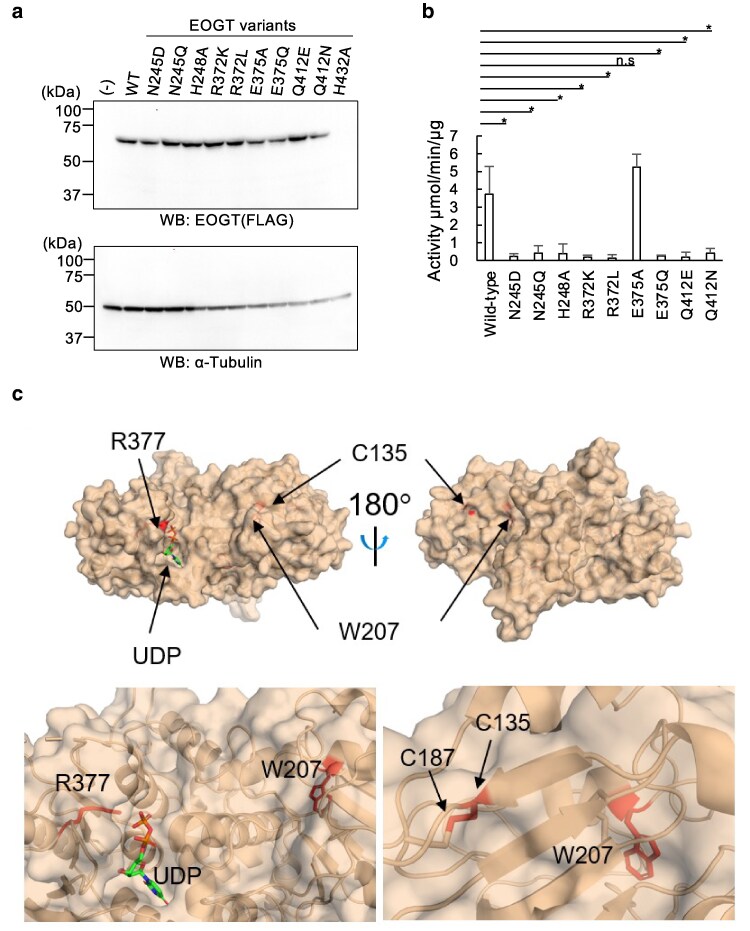
Structural and functional characterization of the UDP-binding site in EOGT and its disease-related mutations. a) Expression levels of EOGT variants carrying substitutions near the UDP-binding site. Wild-type and variant EOGT were expressed in HEK293T cells. The cell lysates were analyzed via SDS–PAGE, and EOGT and α-tubulin were detected using specific antibodies. (−), nontransfected cells. b) *O*-GlcNAc transferase activities of EOGT variants. FLAG-tagged mouse EOGT was incubated with UDP–GlcNAc in an in vitro *O*-GlcNAc transferase assay. Values are expressed as the mean ± SD of biological replicates (*n* = 3–7). Statistically significant differences compared with wild-type EOGT were determined via Tukey's multiple comparison test. *P < 0.01; n.s., not significant. C) Location of AOS-associated *EOGT* mutations (C135, W207, and R377). An enlarged view of the mutation sites (red) is shown.

To validate the contribution of conserved residues to UDP interactions (Fig. 1E), four residues (N245, R372, Q412, and H432) were individually evaluated via site-specific mutagenesis. While the expression of the H432A variant was reduced in HEK293T cells, the other variants showed expression levels comparable to that of wild-type EOGT (Fig. 2a). The purified variant proteins, except for H432A, were analyzed for in vitro *O*-GlcNAc transferase activity using a commercial UDP-Glo assay (Fig. S2). We found that UDP–GlcNAc remained stable during its incubation with EOGT protein alone, ruling out the glycosylation-independent generation of UDP during the UDP-Glo assay (Fig. S3). When *Drosophila* EGF20 (dEGF20) was used as a GlcNAc acceptor substrate, wild-type EOGT exhibited a Michaelis–Menten constant (*Km*) for UDP–GlcNAc of 20.96 ± 8.07 μM (*n* = 5), consistent with a previous report (11). Individual mutations at N245, R372, and Q412 severely diminished enzymatic activity, preventing accurate kinetic measurements (Fig. 2b).

Both N245D (charge-introducing) and N245Q (conservative) mutations markedly abolished enzyme activity. Similarly, R372K (charge-conserving) and R372L (hydrophobic) mutations abolished EOGT activity, suggesting the need for Asn and Arg residues in direct interactions with α and β phosphates of UDP (Fig. 1E). R377 was previously characterized as the R377Q pathogenic mutation associated with AOS (10). The R377Q variant drastically abolished enzymatic activity (11), consistent with our structural analysis showing its direct interaction with the β phosphate moiety of UDP (Figs. 1E and 2c). Additional mutagenesis was performed for E375, positioned at the edge of the UDP-binding pocket, to evaluate the effects of amino acid residues not relevant to UDP binding. The E375A variant, but not E375G, retained enzymatic activity, and the *Km* for UDP–GlcNAc (21.23 ± 2.59 μM; *n* = 3) was comparable to that of wild-type EOGT. Along with the results for the Q412E and Q412N variants, the critical residues mediating UDP interactions observed in the crystal structure were successfully validated via site-directed mutagenesis and enzyme activity assays.

In addition to R377Q, W207S and C135Y variants of EOGT are also linked to AOS (19, 23). In our crystal structure, W207 was buried in the hydrophobic core, suggesting a role in maintaining the structural integrity of EOGT (Fig. 2c). In contrast, C135 formed a disulfide bond with C187 (Fig. 2c), underscoring the critical role of this specific disulfide bond in the enzymatic activity of EOGT. These observations support the pathological relevance of these EOGT mutations.

### Comparative analysis of the UDP-binding pocket in EOGT and POMGNT2

**Figure 3:**
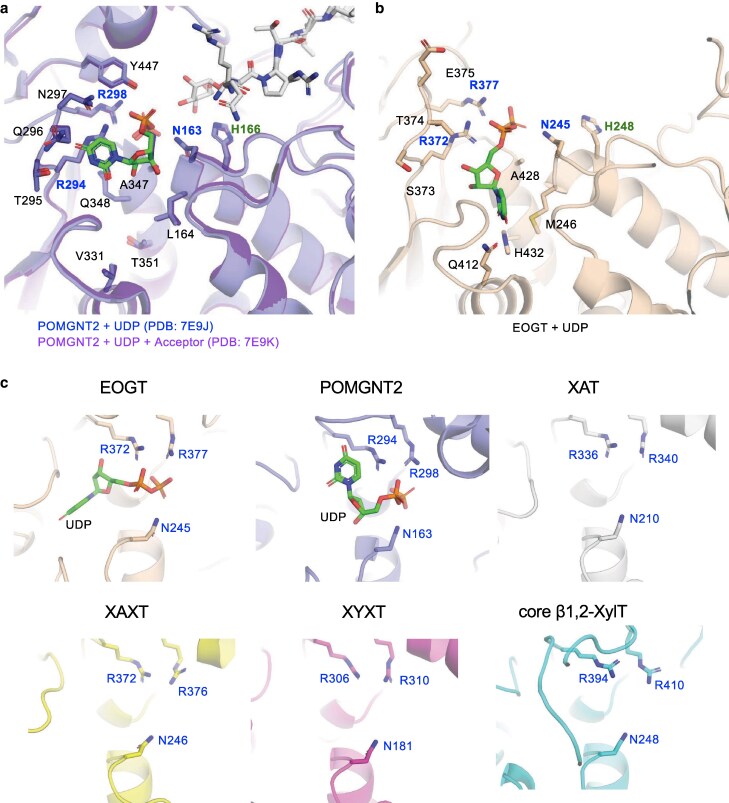
Structural comparison of the N–R–R constellation among representative GT61 enzymes. a) Structure of the UDP-binding pocket in POMGNT2, another enzyme in the GT61 family, based on previously reported crystal structures (*B. taurus* POMGNT2–UDP complex [PDB: 7E9J; blue] and POMGNT2–UDP–acceptor complex [PDB: 7E9K; purple]) (24). Blue, conserved amino acids constituting the N–R–R constellation; green, proposed catalytic residue in POMGNT2. b) Structure of the UDP-binding pocket in EOGT based on the crystal structure of the EOGT–UDP complex. Blue, conserved amino acids constituting the N–R–R constellation; green, His residue corresponding to the proposed catalytic residue in POMGNT2. c) Close-up view of the N–R–R constellation in EOGT, POMGNT2, XAT, XAXT, XYXT, and core β1,2-XylT of the GT61 family. EOGT and POMGNT2 (PDB: 7E9J) are shown in a complex with UDP. The structures of XAT from *Triticum aestivum* (gray), XAXT from *Oryza sativa* (yellow), XYXT (pink), and core β1,2-XylT (light blue) were predicted using AlphaFold2. The gene and reference lists are presented in Tables S1 and S4, respectively.

Another GT61 family member relevant to our structural comparison is POMGNT2 (EC 2.4.1.312), which catalyzes the addition of β1,4-linked GlcNAc to the *O*-mannose residue of α-dystroglycan (18, 19). Because EOGT and POMGNT2 are the only mammalian enzymes reported in the GT61 family and both localize in the ER and use UDP–GlcNAc as a common donor substrate, we hypothesized that the recognition mechanism for UDP could be similar between the two enzymes. Comparison with the crystal structure of the *Bos taurus* POMGNT2–UDP complex (Protein Data Bank: 7E9J) (24, 25) revealed several conserved residues interacting with the α- and β-phosphates of UDP between EOGT and POMGNT2: R372, R377, and N245 in EOGT, which align with R294, R298, and N163 in POMGNT2 (Fig. 3a). A previous study demonstrated that alanine substitution of each of these POMGNT2 residues significantly reduced enzymatic activity in an in vitro GlcNAc-transferase assay, indicating that these conserved residues are required for UDP recognition in POMGNT2 (24). These findings suggest that a conserved triad of one asparagine and two arginine residues is a common mechanism for mediating UDP interactions. Indeed, these residues are highly conserved among POMGNT2 orthologs (Fig. S4).

Despite these similarities, some notable differences were observed. EOGT contained additional residues, Q412 and H432, which directly interacted with the uridine moiety of UDP (Figs. 3b and S4). Consistently, Q412E or Q412N mutation resulted in the loss of enzymatic activity (Fig. 2a and B). In contrast, POMGNT2 lacked the corresponding residues (Fig. 3a), showing structural divergence, leading to different orientations of the uridine moiety in the UDP-binding pocket. Although both enzymes shared a core set of conserved residues critical for UDP interactions, EOGT possessed unique structural elements conferring distinct regulatory properties.

Notably, H248 in EOGT, positioned adjacent to the conserved UDP-binding site, corresponded to H166 in POMGNT2, the proposed catalytic residue directly interacting with the *O*-mannose residue (24). H248 is invariant in metazoan EOGTs (Figs. S1 and S4); however, its functional role remains unclear, warranting further structural elucidation of the EOGT–EGF complex.

### N–R–R constellation in the UDP-binding site is conserved among GT61 family enzymes

We further evaluated the conservation of the identified triad across the GT61 family, which comprises plant glycoprotein β-1,2-xylosyltransferase (core β1,2-XylT) (26), xylan β-1,2-*O*-xylosyltransferase (XYXT) (27), xylan 2-*O*-arabinosyltransferase (XAXT) (28–30), and xylan arabinosyl transferase (XAT) (31) (Fig. 3c; Table S2). Although no information is available on the three-dimensional structures of plant GT61 enzymes bound to UDP, previous studies have performed structural modeling and molecular docking of rice, poplar, and *Sorghum* GT61 enzymes (26–28, 32, 33). These studies predicted that the conserved Asn and Arg residues interact with the phosphate groups of UDP and sugar moieties of UDP–sugar donors. Although the additional roles of sugar moiety interactions remain unknown, previous reports on plant enzymes strongly support a conserved structural framework for UDP recognition across the GT61 family.

**Figure 4:**
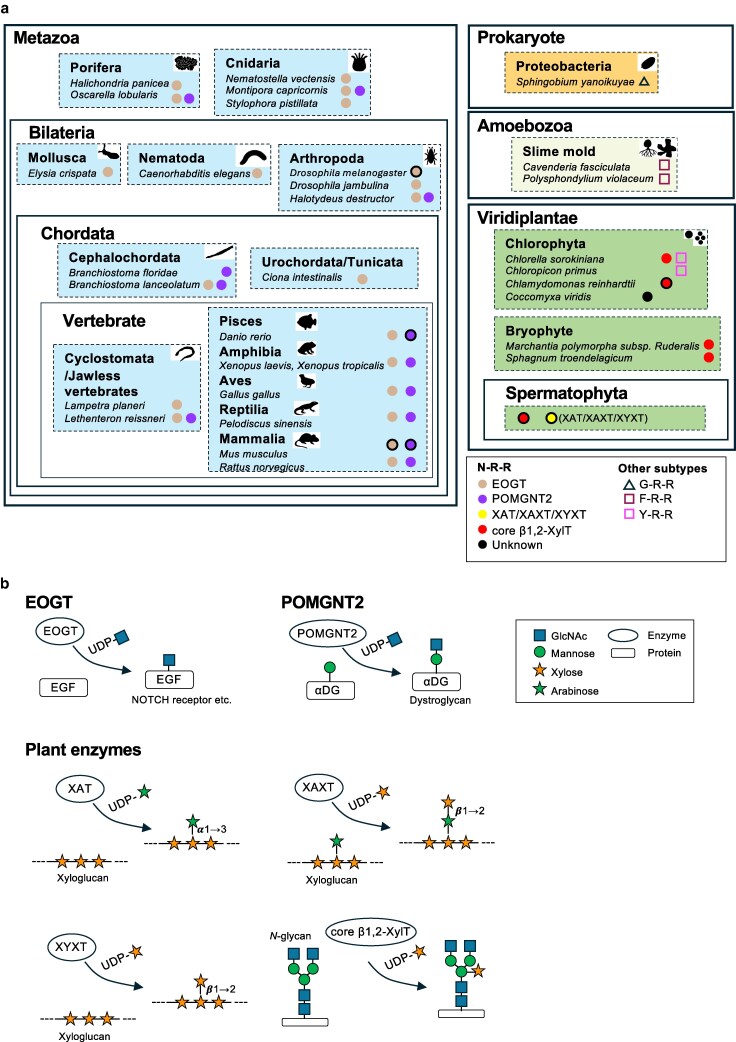
Phylogenetic classification of GT61 proteins based on the N–R–R constellation. a) Classification of GT61 proteins in Metazoa, Viridiplantae, Amoebozoa, and prokaryote organisms based on the presence of the N–R–R or alternative (G–R–R, F–R–R, and Y–R–R) constellations. Proteins shown in this figure are listed in Tables S1–S5. Their predicted UDP-binding pocket structures were modeled using AlphaFold2 (Figs. 3c, 5, and S6–S8). GT61 proteins with experimentally confirmed enzyme activities are indicated by outlined circles. The reference list is presented in Table S2. b) Enzymatic activities of EOGT, POMGNT2, XAT, XYXT, XAXT, and core β1,2-XylT.

These findings led us to designate the triad of asparagine and two arginine residues as the “N–R–R constellation,” a structural element critical for UDP binding in the GT61 family. AlphaFold2-based predictions of EOGT and POMGNT2 orthologs universally conformed to the N–R–R constellation in early-diverging metazoans, including cnidarians and poriferans (Figs. 4 and S5–S8; Tables S1–S5). Similarly, *Spermatophyta* GT61 proteins homologous to core β1,2-XylT and xylan-modifying enzymes and *Bryophyte* core β1,2-XylT orthologs were predicted to conform to the special consensus sequence. These findings collectively suggest that the N–R–R constellation is deeply embedded in the evolutionary history of eukaryotes, reflecting its essential role in enzymatic activity.

### Evolutionary trajectory of the N–R–R constellation

**Figure 5:**
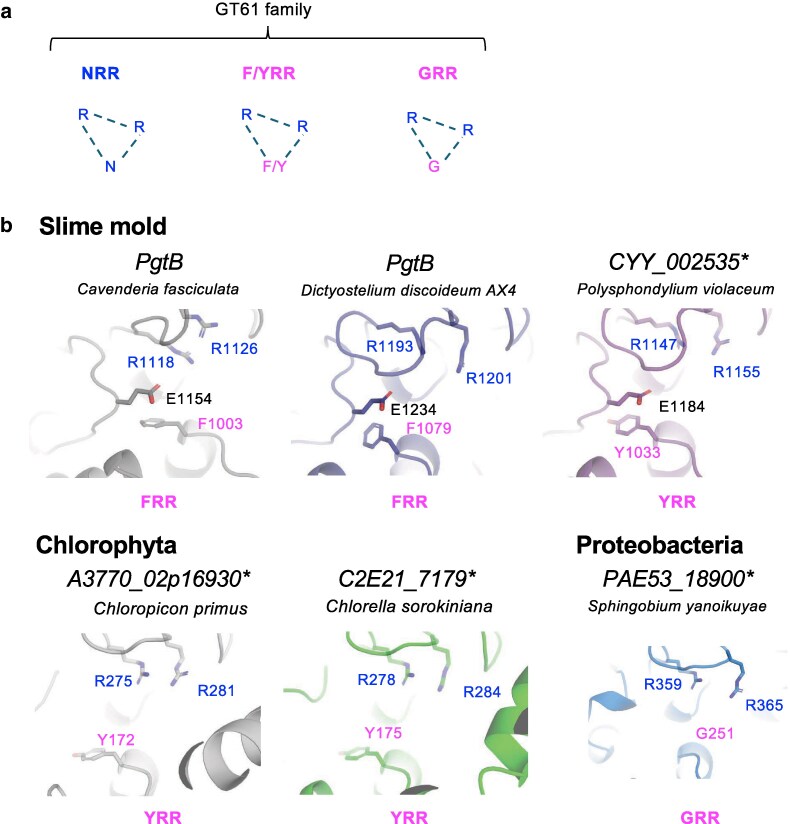
Structural variants of the N–R–R constellation identified across GT61-related proteins. a) Subtypes of the N–R–R constellation. GT61 proteins are classified into three subtypes based on the residues forming the constellation at the UDP-binding site: N–R–R, F/Y–R–R, and G–R–R. b) AlphaFold2-predicted structures of GT61 proteins in slime molds, Chlorophyta, and Proteobacteria. The gene list is presented in Table S5. Blue, conserved N–R–R amino acid residues. Pink, aromatic amino acid and glycine residues.

To explore potential subtypes of the N–R–R constellation, we examined slime molds (Fig. 5; Table S5), which serve as simple model organisms for evolution studies. In *Dictyostelium discoideum*, we identified PgtB, which harbors multiple domains similar to GT1, GT2, GT4, and GT61 (34, 35). AlphaFold2-based structural prediction of the GT61-like domain of PgtB revealed a conserved triad of phenylalanine (F) and two arginine residues (Fig. 5b; Table S5), which is preserved in another slime mold, *Polysphondylium violaceum*. In chlorophytes, a related Y–R–R constellation was found in *Chlorella sorokiniana* and *Chloropicon primus*. The presence of aromatic residues instead of arginine possibly interferes with UDP–sugar donor interactions, suggesting that the F/Y–R–R constellation performs distinct functional roles among GT61-related proteins. Interestingly, in Proteobacteria, only two arginine residues (R–R) were retained, and N or Y/F residues were not conserved (Fig. 5b). These constellations may have arisen independently in multiple lineages, with the N–R–R motif retained as the dominant form during evolution.

## Discussion

The EOGT crystal structure revealed a typical GT-B fold with two Rossmann-like domains. Consistent with this fold, EOGT lacked a canonical DxD motif and instead, the N–R–R constellation serves as the key determinant for UDP recognition. Although EOGT exhibits *O*-GlcNAc transferase activity in the absence of divalent cations, Mn^2+^ enhances its activity (36). In addition, the Arabidopsis core β1,2-XylT exhibits Mn^2+^-enhanced behavior (37, 38) and enzymatic activities of several plant GT61 homologs have been assayed in the presence of Mn^2+^ (20, 39). Since no metal ions were observed in our crystal structure, the precise binding site and mechanistic contribution of Mn^2+^ to GT61 enzyme catalysis remain to be elucidated.

EOGT was modified with *N*-glycans at N263 and N354. Although *N*-glycosylation is dispensable for EOGT enzyme activity, *N*-glycan deficiency results in decreased protein expression and a lack of EOGT at the periphery of the ER (15). Our crystal structure of EOGT revealed the chitobiose moiety of *N*-glycan at N263, which interacted with several conserved amino acid residues. This suggests that *N*-glycans of EOGT contribute to both proper subcompartment distribution in the ER and protein stability, mediated at least in part by the interaction with *N*-glycan at N263.

In addition to *N*-glycan modifications, previous studies implicated the structural features that contribute to EOGT enzymatic function (17). The DYD_295-297_ sequence in EOGT differs fundamentally from the canonical DxD motif that functions as a metal-coordinating donor-binding motif in GT-A-fold GTs. Structural analysis confirms that EOGT adopts a GT-B-fold architecture, and the DYD_295-297_ sequence is positioned distal to the UDP-binding site (Fig. 1). Interestingly, this sequence is conserved in vertebrates but is more diverse across other organisms (Fig. S1), suggesting that it may represent a lineage-specific feature rather than a classical donor-binding motif. The functional role of this sequence remains to be elucidated.

Our structural analysis also revealed a dimeric assembly of EOGT in the crystal. However, size-exclusion chromatography performed during purification for crystallographic analysis showed a single peak corresponding to the monomeric form of EOGT. Therefore, the observed dimer in the crystal structure may represent a crystallization artifact.

Within the catalytic region, H248 appears to play an important role in enzymatic function. EOGT H248 is invariant among GT61 family members (Fig. S1), supporting its functional importance. Alanine substitution at this residue markedly reduced the enzymatic activity of EOGT (Fig. 2b). In the case of POMGNT2, the corresponding residue H166 has been reported to interact with the mannose moiety of the acceptor substrate, α-dystroglycan, and proposed to act as a catalytic base (24). Similarly, Ser/Thr residues of the *O*-GlcNAc modification site of EGF domains could interact with H248, although this possibility requires further investigation.

The conserved N–R–R constellation, despite the diversity of substrates (proteins in animals vs. polysaccharides in plants) for GT61 proteins, led us to analyze the related GT, OGT in the GT41 family. The crystal structure of OGT (7) revealed a UDP-binding site entirely distinct from that of EOGT (Fig. S9A). Similarly, the N–R–R constellation was not evident in other GT families adopting a GT-B fold, suggesting a unique UDP-binding element specific to the GT61 family. Therefore, the presence of N–R–R residues is a possible hallmark of catalytically active GT61 enzymes.

Interestingly, GT61 proteins in Proteobacteria contain two conserved arginine residues but lack the N, Y, or F residues found in metazoan GT61 members. The corresponding position is occupied by glycine, which lacks a side chain. This difference may represent a putative ancestral structural configuration of GT61 enzymes in Proteobacteria (40). As proteins with multiple EGF domains have been reported in Porifera and Cnidaria (41), EOGT possibly co-evolved with the expanding repertoire of EGF domains during early metazoan evolution. Thus, GT61 proteins exemplify a conserved structural framework that enables diversification via substrate-driven selection and incremental refinement of donor-binding residues.

Comparison of the structure of EOGT with those of other EGF domain-modifying enzymes adopting GT-B folds indicated that EOGT possessed a broader acceptor substrate-binding cleft than POFUT1 and POGLUT1 (42–44) (Fig. S9B). Previous comprehensive site-specific *O*-glycan analysis of mouse NOTCH1 has revealed that EOGT-dependent *O*-GlcNAc modification occurs with variable stoichiometry, in contrast to the constantly high stoichiometry reported at the *O*-Fuc and *O*-Glc modification sites catalyzed by POFUT1 and POGLUT1, respectively (45). This differential *O*-GlcNAcylation stoichiometry can be partly explained by local variability in the consensus sequence surrounding the modification site. However, the broader binding face of the EGF domain possibly constitutes an undefined structural element required for efficient *O*-GlcNAcylation by EOGT. Future studies, including the co-crystallization of EOGT and EGF domains or docking simulations using various EGF domains with different *O*-GlcNAcylation stoichiometries, are essential to address this question.

In summary, our study provides insights into the evolution of GT61 enzymes in terms of UDP binding and a rational basis to understand EOGT-related pathogenesis and further elucidate the enzymatic mechanisms of EOGT.

## Materials and methods

### Cell lines

HEK293T cells were obtained from the RIKEN BioResource Research Center (Tsukuba, Ibaraki, Japan). The cells were cultured in Dulbecco's modified Eagle's medium supplemented with heat-inactivated fetal bovine serum and penicillin–streptomycin. To generate HEK293T cells stably expressing FLAG-tagged mouse EOGT, the cells were transfected with pSeqtag2-FLAG-HRV_3C_site-mEOGT^WT^-MycHis_6_ and selected with 800 μg/mL hygromycin B (Fujifilm, Tokyo, Japan).

### Preparation of mouse EOGT for crystal analysis

HEK293T cells stably expressing FLAG-tagged mouse EOGT were collected using trypsin/ethylenediaminetetraacetic acid (EDTA) and lysed in buffer (20 mM Tris-HCl, pH 7.5; 150 mM NaCl; 1 mM EDTA; 1% NP-40) After incubation on ice for 30 min and centrifugation at 15,000 rpm for 30 min at 4 °C, the supernatant was filtered through a 0.45-μm membrane.

The lysate was applied to an anti-FLAG agarose, washed with Tris-NaCl buffer, and eluted with 3× FLAG peptide. The eluted proteins were concentrated, digested with HRV 3C protease at 4 °C overnight, and further purified on a Superdex 200 column (20 mM 4-(2-hydroxyethyl)-1-piperazineethanesulfonic acid [HEPES], pH 7.5; 100 mM NaCl) using the AKTA Prime Plus system (GE Healthcare, Chicago, IL, United States). Fractions containing purified EOGT were identified by sodium dodecyl sulfate–polyacrylamide gel electrophoresis (SDS–PAGE) and Coomassie staining, pooled, and concentrated. Approximately 0.4 mg of EOGT was obtained from 8 × 10^8^ HEK293T cells. Additional experimental details are provided in the Supplementary material.

### Crystallization, data collection, and structure determination of EOGT

Purified EOGT (5–6 mg/mL) was mixed with 5 mM UDP and crystallized by sitting-drop vapor-diffusion at 293 K. Crystals were obtained in 0.1 M Tris-HCl (pH 8.5) and 25% PEG3,350, cryoprotected with 20% ethylene glycol, and flash-frozen. Diffraction data were collected at beamline X06A of the Swiss Light Source (Silligen-PSI, Switzerland) and processed with XDS (46) and AIMLESS (47). Structure determination was performed by molecular replacement using AlphaFold2 model (48), followed by model building in COOT (49) and refinement with REFMAC5 (50) and phenix.refine (51). Model quality was assessed with MOLPROBITY (52). Statistics are summarized in Table 1, and coordinates were deposited in the Protein Data Bank under the accession code pdb_00009wjk. Structural comparisons used SUPERPOSE (53), and figures were prepared in PyMOL (version 2.0; The PyMOL Molecular Graphics System; Schrödinger, LLC, San Diego, CA, United States). Further experimental details are provided in the Supplementary material.

### In vitro *O*-GlcNAc transferase assay

FLAG-tagged mouse EOGT (3 ng) was incubated with UDP–GlcNAc (0, 10, 50, 100, 200, and 400 μM) and dEGF20 (500 ng) in 25 mM HEPES–NaOH buffer (pH 7.0) with 1 mM MgCl_2_ at 37 °C for 30 min. To quantify the reaction activity, UDP-Glo Glycosyltransferase Assay (Promega, Madison, WI, United States) was performed to measure the amount of reacted UDP, according to the manufacturer's instructions. *Km* was calculated using GraphPad Prism (GraphPad Software, San Diego, CA, United States).

### Statistical analyses

Statistical significance of the differences between wild-type and variant EOGT enzyme activities was evaluated via Tukey's multiple comparison test with a significance level set at *P* < 0.01.

## Supplementary Material

pgag115_Supplementary_Data

## Data Availability

Structural factors and coordinates of the EOGT–UDP complex have been deposited in the Worldwide Protein Data Bank under accession code pdb_00009wjk.
