# Supplementary material for: Crystal structure of epidermal growth factor domain-specific O-linked N-acetylglucosamine transferase reveals a conserved N–R–R constellation for uridine diphosphate recognition in the GT61 family
Source: PNAS Nexus. 2026 Apr 15;5(4):pgag115. doi: 10.1093/pnasnexus/pgag115 (PMC13123520; doi:10.1093/pnasnexus/pgag115)
Supplement: pgag115_Supplementary_Data [file pgag115_supplementary_data.pdf]

Supporting Information for
Crystal structure of EOGT reveals a conserved N–R–R constellation for UDP recognition in the GT61 family

Yuko Tashima[a,b,1], Masamichi Nagae[c,d,1,*], Jiaoyang Jiang[e], and Tetsuya Okajima[a,b,*]

[a]Department of Molecular Biochemistry, Nagoya University Graduate School of Medicine, 65 Tsurumai-Cho, Showa-Ku, Nagoya, Aichi, 466-8550, Japan
[b]Institute for Glyco-core Research (iGCORE), Nagoya University, Furo-Cho, Chikusa-Ku, Nagoya, Aichi, 464-8601, Japan
[c]Department of Molecular Immunology, Research Institute for Microbial Diseases, The University of Osaka, Suita, Osaka, 565-0871, Japan
[d]Laboratory of Molecular Immunology, Immunology Frontier Research Center, The University of Osaka, Suita, Osaka, 565-0871, Japan
[e]Pharmaceutical Sciences Division, School of Pharmacy, University of Wisconsin-Madison, Madison, WI 53705, USA

[*]Corresponding authors:
Masamichi Nagae, Ph.D.
Assistant Professor
Department of Molecular Immunology, Research Institute for Microbial Diseases, The University of Osaka, Suita, Osaka, 565-0871, Japan
Tel.: +81-6-6879-8307; Fax: +81-6-6879-8308; Email: mnagae@biken.osaka-u.ac.jp
Tetsuya Okajima, MD, Ph.D.
Professor
Department of Biochemistry II, Nagoya University Graduate School of Medicine, 65 Tsurumai-Cho, Showa-Ku, Nagoya, Aichi, 466-8550, Japan
Tel.: +81-52-744-2068; Fax: +81-52-744-2069; Email: tokajima@med.nagoya-u.ac.jp

This PDF file includes:

Figures S1 to S9
Tables S1 to S6
Supporting Materials and Methods

[1]These authors contributed equally to this work.

Supporting Figures

A

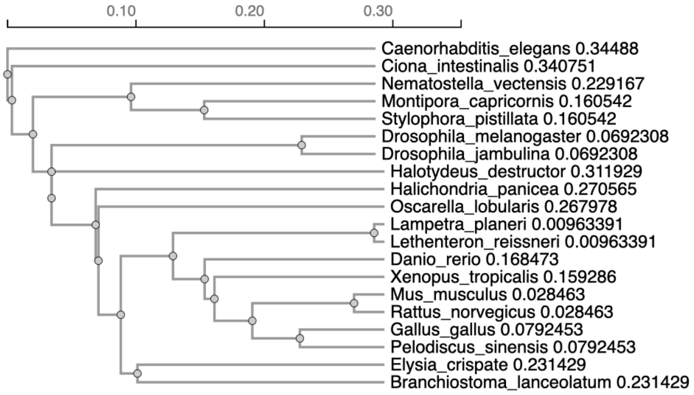

B

```
                                                      AOS
                                                      C135                 R155
Caenorhabditis_elegans      VADLFFKQGDFGYLESRLVKHD---ICTSD--HVSLSTLSCSDDLTHCVGTNIFFDFSNL   143
Ciona_intestinalis          FKQDFWKMVDFGYVEERRKEFKELLICQPH--EDQASSLQCSKYTRYCVARNLYVDFTNL   122
Nematostella_vectensis      KVNSFWTEADFGYVKKFIDSTKD--VCKSD--KVNGGSLQCSKELGYCTANNLYMDLRKG   160
Montipora_capricornis       KVYQFWKDADFGYVKSVQDSLQF--VCKPKGTKADSSSLVCSKQLTYCRAQNLYLDLTDS   155
Stylophora_pistillata       KIYHFWKESDFGFVKSVQDSLQL--VCKPKEYQADGSSLVCSKQLTYCKAKHLFMDFRSM   157
Drosophila_melanogaster     QVRTFYNQADFGYIQEQLSQLTP--QCVPT--YLGDSSLECTHYLRFCRGRNLLFDFRGL   150
Drosophila_jambulina        QVKTFYQQADFGYIQEQLAELAP--QCVPT--YLGDSSLECTHYLRFCRGRNLLFDFRDL   159
Halotydeus_destructor       QVDIFDGQADFKYISERRKELKT--YCKAN--TKTGSTLECTSHARLCRGKKIAIDFSGL   147
Halichondria_panicea        KAEDFWKQGDFGYVKKEMDSLTT--LCKPQ--VKGDSMLKCSENMRYCRARNLYIDFRRF   144
Oscarella_lobularis         KVERFFDSADFGWVKTIRSEMTT--YCKSK--GVGGSSLRCGDLLRHCRAINIYFDLRAI   140
Lampetra_planeri            AEYLYWRQADFGYVKERVGELKT--LCRAR--QPGDSSLTCVQYTRYCRATNLYVDLRKP   209
Lethenteron_reissneri       AEYLYWRQADFGYVKERLGELKT--LCRAR--QPGDSSLTCVQYTRYCRATNLYVDLRKP   158
Danio_rerio                 AQELFWKQADFGYVRERLNEMKT--LCKPS--KPADSSLKCSSHMRFCRATNLYLDLRSP   158
Xenopus_tropicalis          SQEVFWKQADFGYVKERLAETQI--LCRPQ--EQGDSMLACSRNLQHCRATNLYLDLRNP   164
Mus_musculus                AQDMFWRQADFGYARERLGEIRT--ICQPE--RASDSSLVCSRYLQYCRATGLYLDLRNI   166
Rattus_norvegicus           AQDMFWKQADFGYARERLEEIRM--FCRPE--SASDSSLLCSRYLQYCRATGLYLDLRNI   166
Gallus_gallus               AQQIFWKQADFGYIRERLNEMKT--HCKPT--VTGDSSLTCSQFLQHCRATNLYIDLRTA   169
Pelodiscus_sinensis         AQQIFWKQADFGYVKERLDEMKT--HCKPI--TKGDSSLACSQYLQHCRATNLYIDLRAI   166
Elysia_crispate             QEYKFWSTADFGMLLERKKELRM--YCKPN--SKEDSSLQCTKYLRYCRAANIYLDFSSK   165
Branchiostoma_lanceolatum   TADLFWKEADFGYVWERLQEMTV--MCKAQ--EEGDSSLSCVKHLRYCRATNIYMDFINL   154
                                :    **        .      *         . * *      * .  : .*:

                                                                            AOS
                                                                            W207
Caenorhabditis_elegans      NI-----KTSTRYRQDVIQAGRV-GGKCSNFDEKVLKQNSNVKGYLMSWADELQNFRSSS   197
Ciona_intestinalis          MQ----ANNRDKFRENIFKPGQI-GGDCEVNEKMLEDQNEH-KSALQSWYAELETFTKVD   176
Nematostella_vectensis      ------T----RTQNERFNPEML-AGFCDLDLAVLQDLGGH-RMELTSWVAEIEKYQSIP   208
Montipora_capricornis       ------VQSHDRSQTNLFKPKVI-GGYCDVDSNTLTEDGKY-RMELSSWLNELEDFSQLS   207
Stylophora_pistillata       ------PNGQSTSKSDKLKPQKI-GGFCEVDASALSEEGKY-RMELSSWLNEVEDFSSLP   209
Drosophila_melanogaster     EQ----REERIRYHMDVLGPGQL-LGHCKLNRTRLSGEMEHIGSALQSWGPELRNFDVLP   205
Drosophila_jambulina        AQ----REERIRYHMDVLKPGQL-LAHCELNRTRLAGEMDHIGSALQSWGPELRNLDVLP   214
Halotydeus_destructor       IG----IKEPMKYSGDVLSDGQVMAAQCDYDKESFSKLGAH-KSPLQSWFEEMEHFTVV-   201
Halichondria_panicea        KEMSKTEGKYKSFLEDIFQEGEV-GGHCDLNSDLLRSQGEH-KSPLQSWYAELERYTNLT   202
Oscarella_lobularis         SF----ENSRQRFRENVFGDGQV-GGDCELDRDALAKQGAH-RSALQSWYGELERYTSLP   194
Lampetra_planeri            ------RRGTDRFSEDFFEAGEI-GGRCDLDAAALAAEGEH-KSPLQSWFAELQSFTQLE   261
Lethenteron_reissneri       ------RRGTDRFSEDFFEAGEI-GGRCDLDADALAAEGEH-KSPLQSWFAELQSFTQLE   210
Danio_rerio                 ------RRGHERYKEDFLEQGEI-GGHCSLNSKALEAEGTH-KSPLQSWFSELQTYSELD   210
Xenopus_tropicalis          ------RRGQENFKEDFLQEGEI-GGRCNLDKQALLSQGAW-KSPLQSWFAELQSYSSLT   216
Mus_musculus                ------KRNHDRFKEDFLQGGEI-GGYCKLDSHALVSEGQR-KSPLQSWFAELQGYTQLN   218
Rattus_norvegicus           ------KRNHDRFKEDFLQGGDI-GGYCKLDRHALVSEGQR-KSPLQSWFAELQGYTQLN   218
Gallus_gallus               ------KRNHERFKEDFFQKGEI-GGHCTLDVKAFLAEGQR-KSPLQSWFAELQTFTSLN   221
Pelodiscus_sinensis         ------KRNHDRFKEDFLPNGQI-GGHCNLDSQAFMAEGQR-KSPLQSWFAELQTYTPLS   218
Elysia_crispate             PITK--SNERDRYREDVLGPGLV-GGHCELDDVNLRAQGEH-KSPLQSWFAEIQHFSSLE   221
Branchiostoma_lanceolatum   --DS--ENNNNRYRENLFNAGQI-GGHCKLYPTRLSAEGKH-KSALQSWYAELEMFSSLP   208
                                           : :    :  . *      .          * **   *:.
```

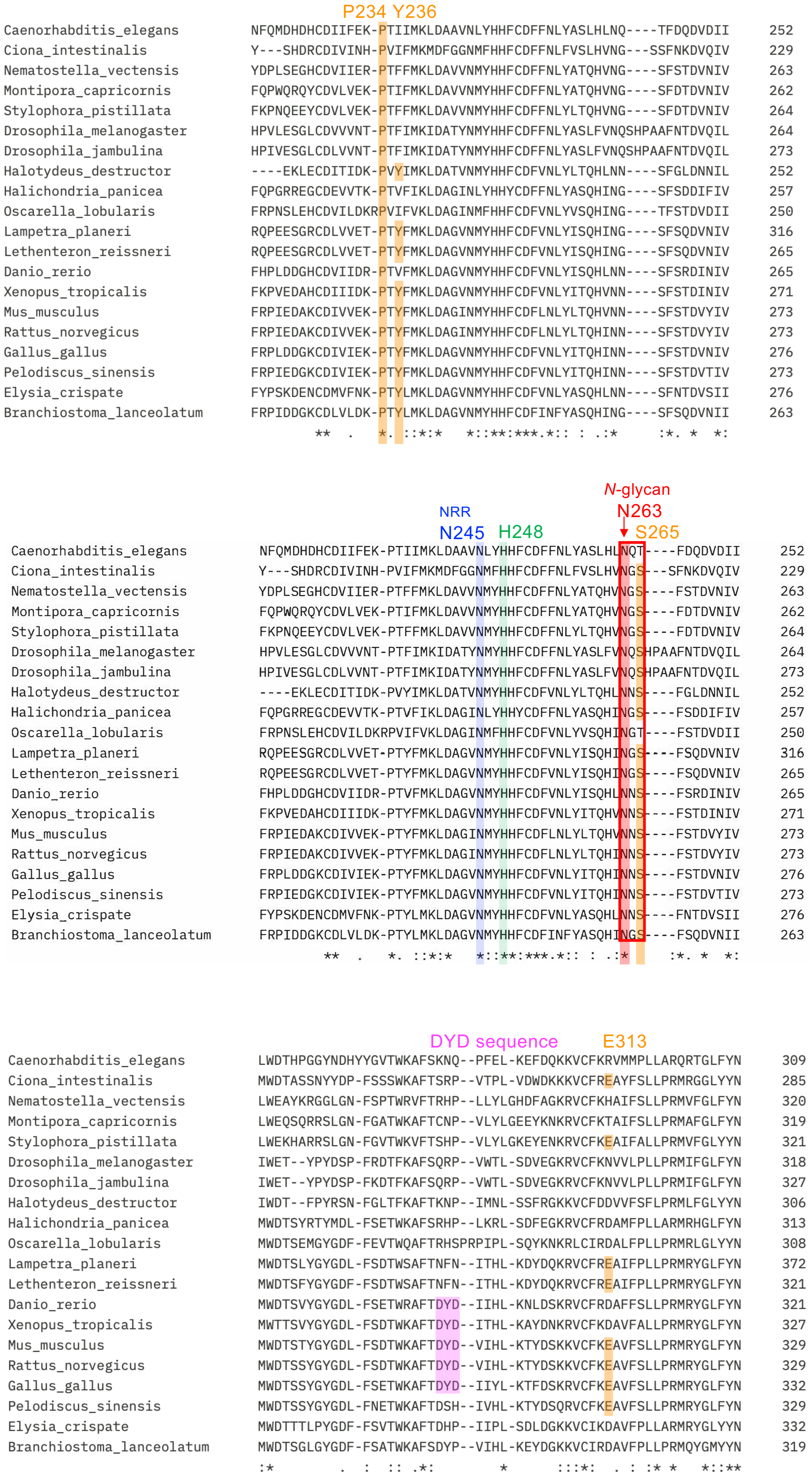
P234 Y236
Caenorhabditis_elegans NFQMDHDHCDIIFEK-PTIIMKLDAAVNLYHHFCDFFNLYASLHLNQ----TFDQDVDII 252
Ciona_intestinalis Y---SHDRCDIVINH-PVIFMKMDFGGNMFHHFCDFFNLFVSLHVNG---SSFNKDVQIV 229
Nematostella_vectensis YDPLSEGHCDVIIER-PTFFMKLDAVVNMYHHFCDFFNLYATQHVNG----SFSTDVNIV 263
Montipora_capricornis FQPWQRQYCDVLVEK-PTIFMKLDAVVNMYHHFCDFFNLYATQHVNG----SFDTDVNIV 262
Stylophora_pistillata FKPNQEEYCDVLVEK-PTFFMKLDAVVNMYHHFCDFFNLYLTQHVNG----SFDTDVNIV 264
Drosophila_melanogaster HPVLESGLCDVVVNT-PTFIMKIDATYNMYHHFCDFFNLYASLFVNQSHPAAFNTDVQIL 264
Drosophila_jambulina HPIVESGLCDLVVNT-PTFIMKIDATYNMYHHFCDFFNLYASLFVNQSHPAAFNTDVQIL 273
Halotydeus_destructor ----EKLECDITIDK-PVYIMKLDATVNMYHHFCDFVNLYLTQHLNN----SFGLDNNIL 252
Halichondria_panicea FQPGRREGCDEVVTK-PTVFIKLDAGINLYHHYCDFFNLYASQHING----SFSDDIFIV 257
Oscarella_lobularis FRPNSLEHCDVILDKRPVIFVKLDAGINMFHHFCDFVNLYVSQHING----TFSTDVDII 250
Lampetra_planeri RQPEESGRCDLVVET-PTYFMKLDAGVNMYHHFCDFVNLYISQHING----SFSQDVNIV 316
Lethenteron_reissneri RQPEESGRCDLVVET-PTYFMKLDAGVNMYHHFCDFVNLYISQHING----SFSQDVNIV 265
Danio_rerio FHPLDDGHCDVIIDR-PTVFMKLDAGVNMYHHFCDFVNLYISQHLNN----SFSRDINIV 265
Xenopus_tropicalis FKPVEDAHCDIIIDK-PTYFMKLDAGVNMYHHFCDFVNLYITQHVNN----SFSTDINIV 271
Mus_musculus FRPIEDAKCDIVVEK-PTYFMKLDAGINMYHHFCDFLNLYLTQHVNN----SFSTDVYIV 273
Rattus_norvegicus FRPIEDAKCDIVVEK-PTYFMKLDAGINMYHHFCDFLNLYLTQHINN----SFSTDVYIV 273
Gallus_gallus FRPLDDGKCDIVIEK-PTYFMKLDAGVNMYHHFCDFVNLYITQHINN----SFSTDVNIV 276
Pelodiscus_sinensis FRPIEDGKCDIVIEK-PTYFMKLDAGVNMYHHFCDFVNLYITQHINN----SFSTDVTIV 273
Elysia_crispate FYPSKDENCDMVFNK-PTYLMKLDAGVNMYHHFCDFVNLYASQHLNN----SFNTDVSII 276
Branchiostoma_lanceolatum FRPIDDGKCDLVLDK-PTYLMKLDAGVNMYHHFCDFINFYASQHING----SFSQDVNII 263
** . *. ::*:* *::**:***.*:: : .:* :*. * *:
N-glycan
NRR
N263
N245 H248 S265
Caenorhabditis_elegans NFQMDHDHCDIIFEK-PTIIMKLDAAVNLYHHFCDFFNLYASLHLNQT----FDQDVDII 252
Ciona_intestinalis Y---SHDRCDIVINH-PVIFMKMDFGGNMFHHFCDFFNLFVSLHVNGS---SFNKDVQIV 229
Nematostella_vectensis YDPLSEGHCDVIIER-PTFFMKLDAVVNMYHHFCDFFNLYATQHVNGS----FSTDVNIV 263
Montipora_capricornis FQPWQRQYCDVLVEK-PTIFMKLDAVVNMYHHFCDFFNLYATQHVNGS----FDTDVNIV 262
Stylophora_pistillata FKPNQEEYCDVLVEK-PTFFMKLDAVVNMYHHFCDFFNLYLTQHVNGS----FDTDVNIV 264
Drosophila_melanogaster HPVLESGLCDVVVNT-PTFIMKIDATYNMYHHFCDFFNLYASLFVNQSHPAAFNTDVQIL 264
Drosophila_jambulina HPIVESGLCDLVVNT-PTFIMKIDATYNMYHHFCDFFNLYASLFVNQSHPAAFNTDVQIL 273
Halotydeus_destructor ----EKLECDITIDK-PVYIMKLDATVNMYHHFCDFVNLYLTQHLNNS----FGLDNNIL 252
Halichondria_panicea FQPGRREGCDEVVTK-PTVFIKLDAGINLYHHYCDFFNLYASQHINGS----FSDDIFIV 257
Oscarella_lobularis FRPNSLEHCDVILDKRPVIFVKLDAGINMFHHFCDFVNLYVSQHINGT----FSTDVDII 250
Lampetra_planeri RQPEESGRCDLVVET-PTYFMKLDAGVNMYHHFCDFVNLYISQHINGS----FSQDVNIV 316
Lethenteron_reissneri RQPEESGRCDLVVET-PTYFMKLDAGVNMYHHFCDFVNLYISQHINGS----FSQDVNIV 265
Danio_rerio FHPLDDGHCDVIIDR-PTVFMKLDAGVNMYHHFCDFVNLYISQHLNNS----FSRDINIV 265
Xenopus_tropicalis FKPVEDAHCDIIIDK-PTYFMKLDAGVNMYHHFCDFVNLYITQHVNNS----FSTDINIV 271
Mus_musculus FRPIEDAKCDIVVEK-PTYFMKLDAGINMYHHFCDFLNLYLTQHVNNS----FSTDVYIV 273
Rattus_norvegicus FRPIEDAKCDIVVEK-PTYFMKLDAGINMYHHFCDFLNLYLTQHINNS----FSTDVYIV 273
Gallus_gallus FRPLDDGKCDIVIEK-PTYFMKLDAGVNMYHHFCDFVNLYITQHINNS----FSTDVNIV 276
Pelodiscus_sinensis FRPIEDGKCDIVIEK-PTYFMKLDAGVNMYHHFCDFVNLYITQHINNS----FSTDVTIV 273
Elysia_crispate FYPSKDENCDMVFNK-PTYLMKLDAGVNMYHHFCDFVNLYASQHLNNS----FNTDVSII 276
Branchiostoma_lanceolatum FRPIDDGKCDLVLDK-PTYLMKLDAGVNMYHHFCDFINFYASQHINGS----FSQDVNII 263
** . *. ::*:* *::**:***.*:: : .:* :*. * *:
DYD sequence E313
Caenorhabditis_elegans LWDTHPGGYNDHYYGVTWKAFSKNQ--PFEL-KEFDQKKVCFKRVMMPLLARQRTGLFYN 309
Ciona_intestinalis MWDTASSNYYDP-FSSSWKAFTSRP--VTPL-VDWDKKKVCFREAYFSLLPRMRGGLYYN 285
Nematostella_vectensis LWEAYKRGGLGN-FSPTWRVFTRHP--LLYLGHDFAGKRVCFKHAIFSLLPRMVFGLFYN 320
Montipora_capricornis LWEQSQRRSLGN-FGATWKAFTCNP--VLYLGEEYKNKRVCFKTAIFSLLPRMAFGLFYN 319
Stylophora_pistillata LWEKHARRSLGN-FGVTWKVFTSHP--VLYLGKEYENKRVCFKEAIFALLPRMVFGLYYN 321
Drosophila_melanogaster IWET--YPYDSP-FRDTFKAFSQRP--VWTL-SDVEGKRVCFKNVVLPLLPRMIFGLFYN 318
Drosophila_jambulina IWET--YPYDSP-FKDTFKAFSQRP--VWTL-SDVEGKRVCFKNVVLPLLPRMIFGLFYN 327
Halotydeus_destructor IWDT--FPYRSN-FGLTFKAFTKNP--IMNL-SSFRGKKVCFDDVVFSFLPRMLFGLYYN 306
Halichondria_panicea MWDTSYRTYMDL-FSETWKAFSRHP--LKRL-SDFEGKRVCFRDAMFPLLARMRHGLFYN 313
Oscarella_lobularis MWDTSEMGYGDF-FEVTWQAFTRHSPRPIPL-SQYKNKRLCIRDALFPLLPRMRLGLYYN 308
Lampetra_planeri MWDTSLYGYGDL-FSDTWSAFTNFN--ITHL-KDYDQKRVCFREAIFPLLPRMRYGLFYN 372
Lethenteron_reissneri MWDTSFYGYGDF-FSDTWSAFTNFN--ITHL-KDYDQKRVCFREAIFPLLPRMRYGLFYN 321
Danio_rerio MWDTSVYGYGDL-FSETWRAFTDYD--IIHL-KNLDSKRVCFRDAFFSLLPRMRYGLFYN 321
Xenopus_tropicalis MWTTSVYGYGDL-FSDTWKAFTDYD--ITHL-KAYDNKRVCFKDAVFALLPRMRYGLFYN 327
Mus_musculus MWDTSTYGYGDL-FSDTWKAFTDYD--VIHL-KTYDSKKVCFKEAVFSLLPRMRYGLFYN 329
Rattus_norvegicus MWDTSSYGYGDL-FSDTWKAFTDYD--VIHL-KTYDSKKVCFKEAVFSLLPRMRYGLFYN 329
Gallus_gallus MWDTSSYGYGDL-FSETWKAFTDYD--IIYL-KTFDSKRVCFKEAVFSLLPRMRYGLFYN 332
Pelodiscus_sinensis MWDTSSYGYGDL-FNETWKAFTDSH--IVHL-KTYDSQRVCFKEAVFSLLPRMRYGLFYN 329
Elysia_crispate MWDTTTLPYGDF-FSVTWKAFTDHP--IIPL-SDLDGKKVCIKDAVFPLLARMRYGLYYN 332
Branchiostoma_lanceolatum MWDTSGLGYGDF-FSATWKAFSDYP--VIHL-KEYDGKKVCIRDAVFPLLPRMQYGMYYN 319
:* . : :: .*: * :::*: . : :* * *::**

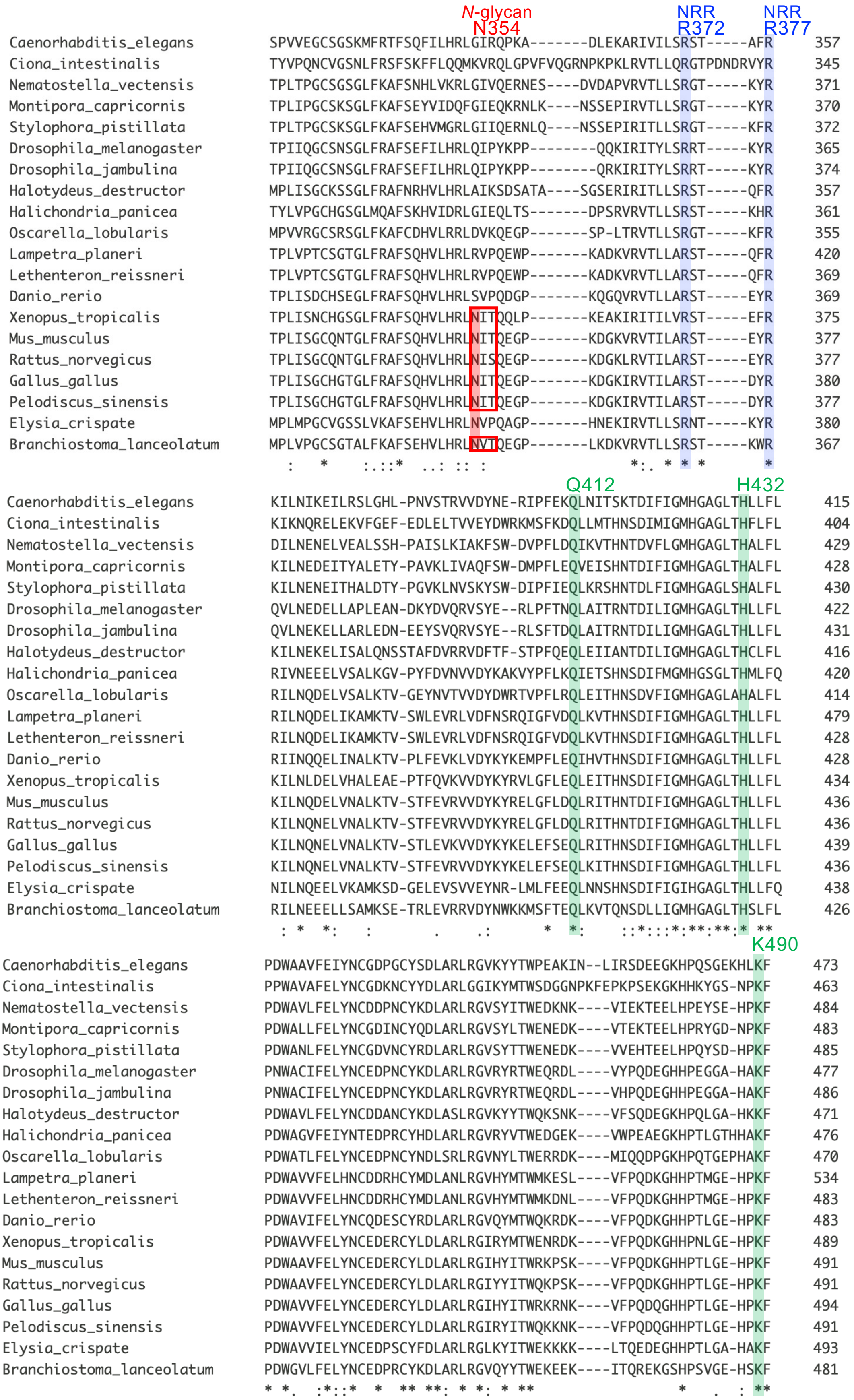

Fig. S1. Multiple sequence alignment of the uridine diphosphate (UDP)-interacting sites of epidermal growth factor (EGF) domain-specific *O*-linked *N*-acetylglucosamine (GlcNAc) transferase (EOGT) for evolutionary validation. Amino acid sequences of EOGT were aligned using the Clustal Omega sequence alignment tool (v1.2.4). (A) Phylogram of evolutionary distances. Evolutionary distances, based on sequence divergence, are shown. Evolutionary distances are indicated as substitutions per site. (B) Sequences represent EOGT homologs with >40% query coverage identified using the BLAST2 alignment tool (listed in Table S3). *Blue*, N–R–R constellation sites (N245, R372, and R377); *red*, *N*-glycosylation sites (N263 and N354); *magenta*, Asp–Tyr–Asp (DYD) sequence; *green*, residues in or around the UDP-interacting sites (Q412, H432, K490, and H248); *orange*, amino acid residues interacting with the chitobiose moiety of *N*-glycan at N263 (R155, P234, Y236, S265, and E313); *brown*, residues corresponding to the mutations (C135 and W207) associated with the Adams–Oliver syndrome (AOS); *red* square, N–X–S/T sequons for *N*-glycosylation. The amino acid sequence surrounding N263 was manually adjusted to better illustrate the N–X–S/T sequon.

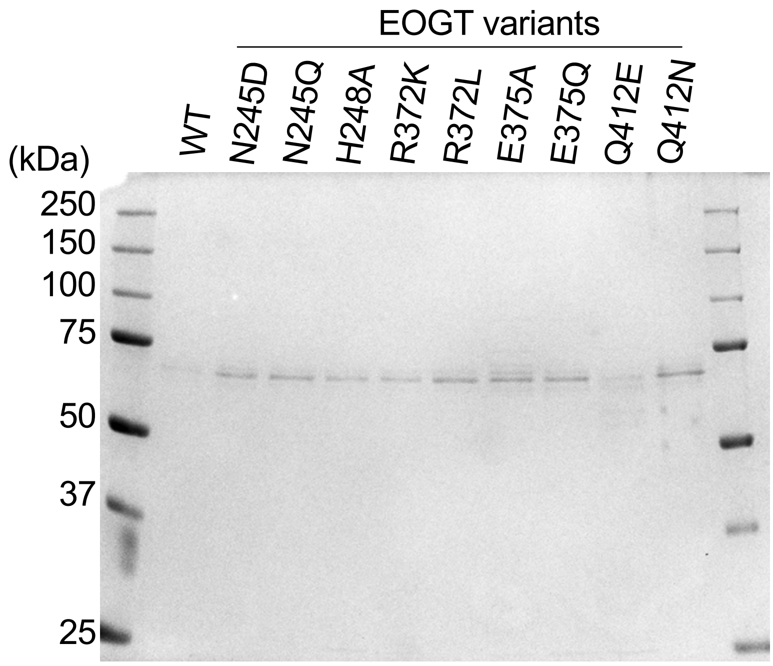

Fig. S2. Coomassie brilliant blue (CBB) blot of purified wild-type (WT) and variant EOGT used in the *in vitro O*-GlcNAc transferase assay.

FLAG-tagged mouse EOGT (WT or variant) was expressed in human embryonic kidney (HEK)-293T cells, purified with anti-FLAG agarose beads, and eluted with the FLAG peptide. The eluate was separated using SDS–PAGE and stained with CBB.

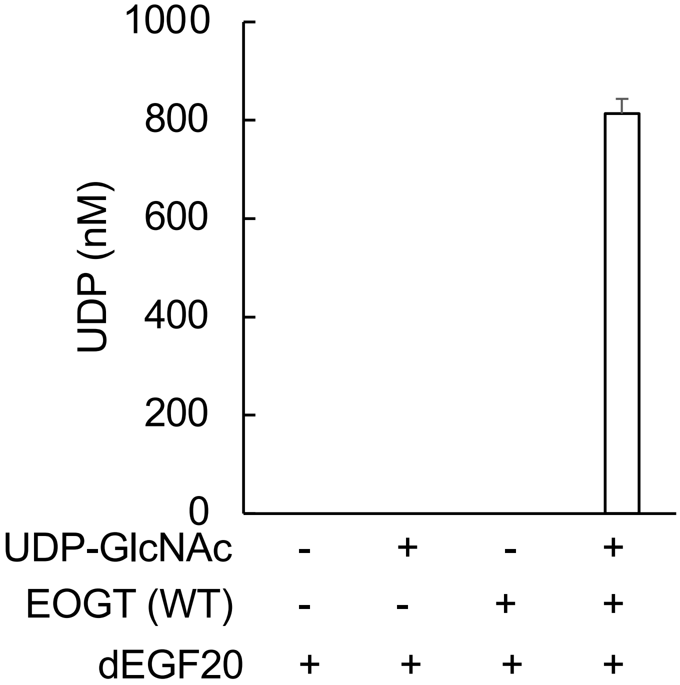

Fig. S3. UDP is not decomposed in the *in vitro* *O*-GlcNAc transferase assay.
FLAG-tagged mouse EOGT (WT) was incubated with 100 μM UDP–GlcNAc and 500 ng dEGF20 in 25 mM HEPES–NaOH (pH 7.0) buffer with 1 mM $MgCl_2$ at 37 °C for 30 min. To quantify the reaction activity, a UDP-Glo glycosyltransferase (GT) assay was performed to measure the amount of UDP, following the instructions. The experiment was performed in triplicate. Error bars represent the standard deviations (n = 3).

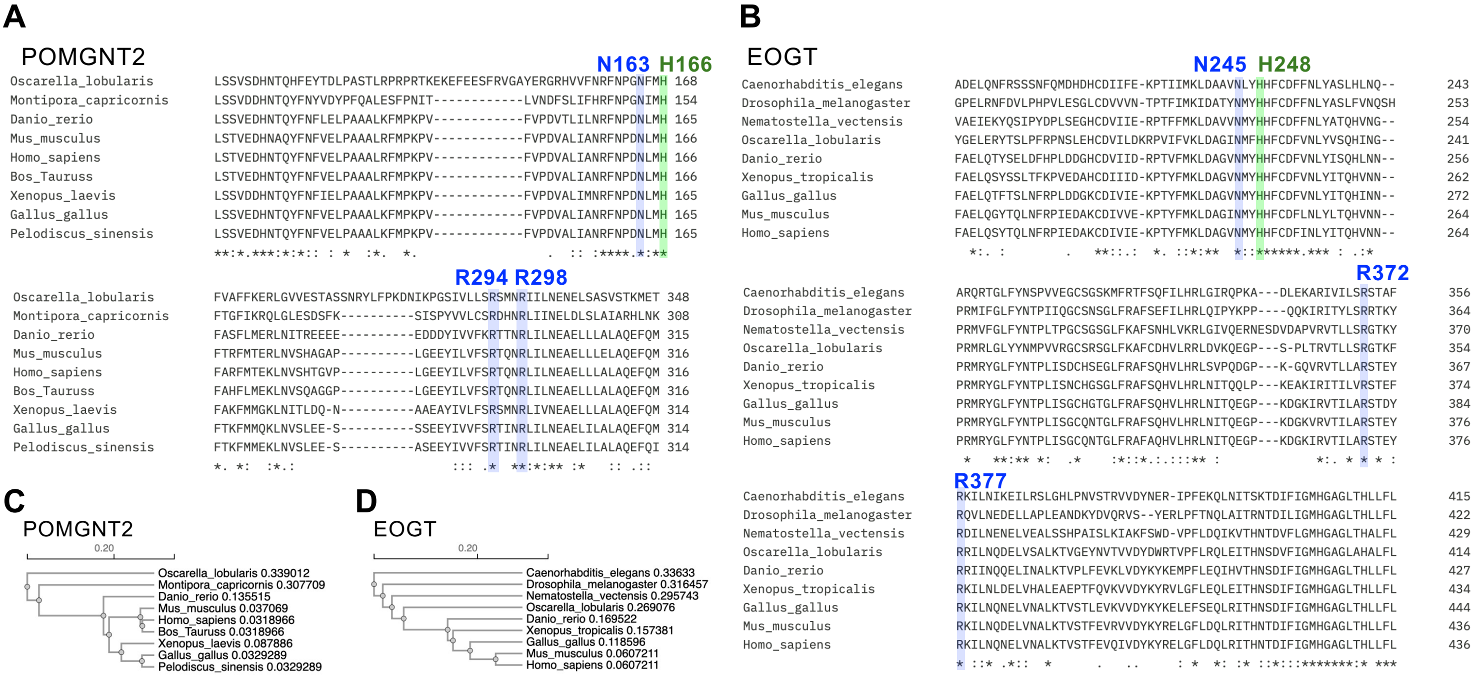

Fig. S4. Alignment of the amino acid residues in the UDP-interacting sites of POMGNT2 and EOGT from representative species.

(A, B) Alignment of the amino acid residues in the UDP-interacting sites of POMGNT2 (panel A), including N163, H166, R298, and R294, and of EOGT (panel B), including N245, H248, R372, and R377. These species correspond to those shown in Fig. S1. Multiple sequence alignments were performed using the Clustal Omega sequence alignment tool (v1.2.4). *Blue*, conserved amino acids constituting the N–R–R constellation; *green*, His residue corresponding to the proposed catalytic residue.

(C,D) Phylogenetic tree based on the sequence alignment shown in panels A and B. Evolutionary distances are indicated as substitutions per site.

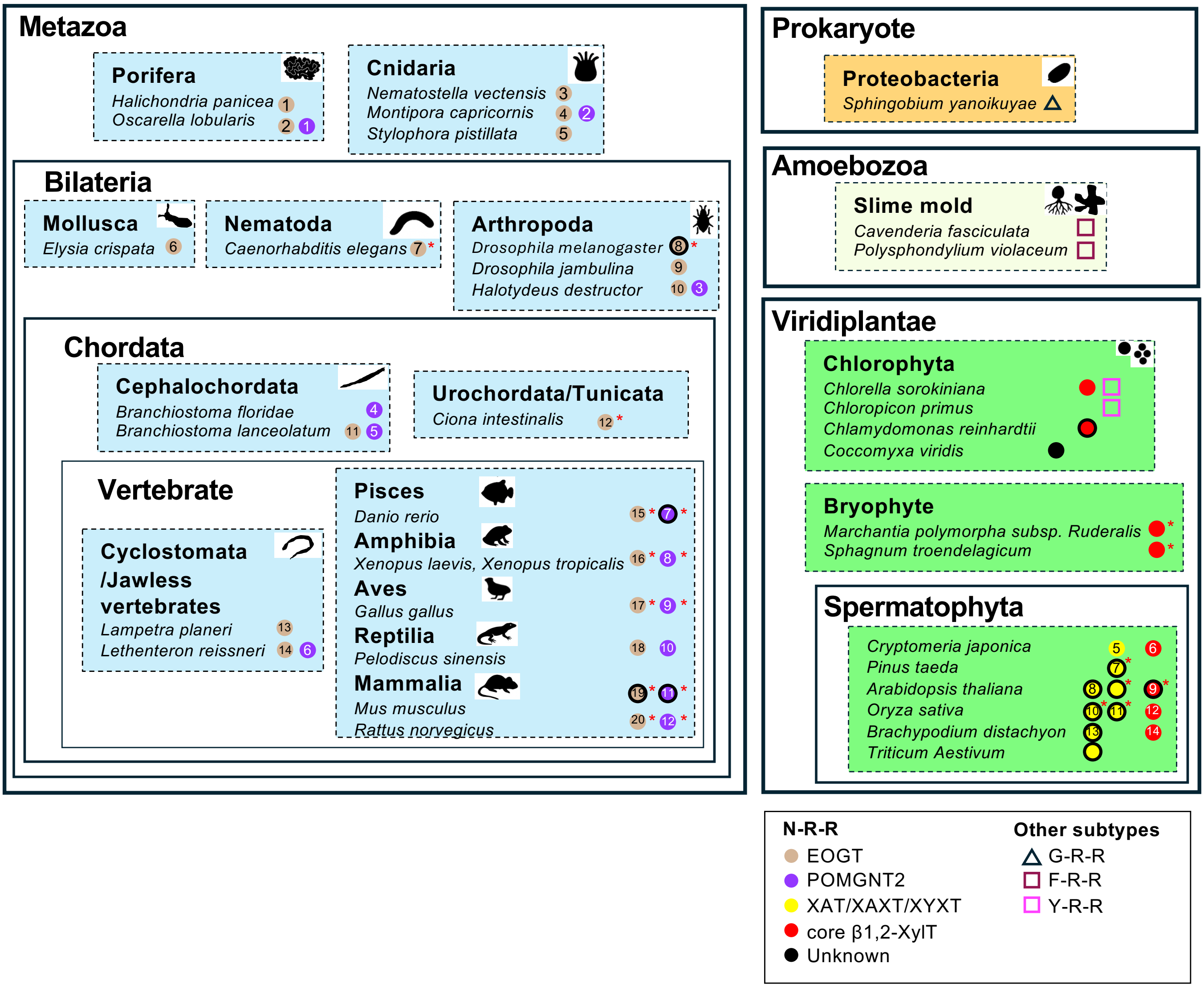

Fig. S5. Information related to Figure 4A.
This figure provides supporting information for Figure 4A, with reference numbers indicating the respective AlphaFold2 models shown in Figs. S6–S8; others without numbers are shown in Figures 3B and 5. The proteins shown in this figure are presented in Tables S1, S2, S4, and S5. GT61 proteins with experimentally confirmed enzyme activities are indicated by outlined circles. The reference list is presented in Table S4. Asterisks indicate the enzymes or orthologs classified in the GT61 family of the Carbohydrate-Active Enzymes (CAZy) database. The list excludes the EOGT ortholog in *Branchiostoma floridae* (XP_035677618.1) because it lacks the N–R–R constellation and adopts a GT-A fold, making it unlikely to function as an active EOGT enzyme.

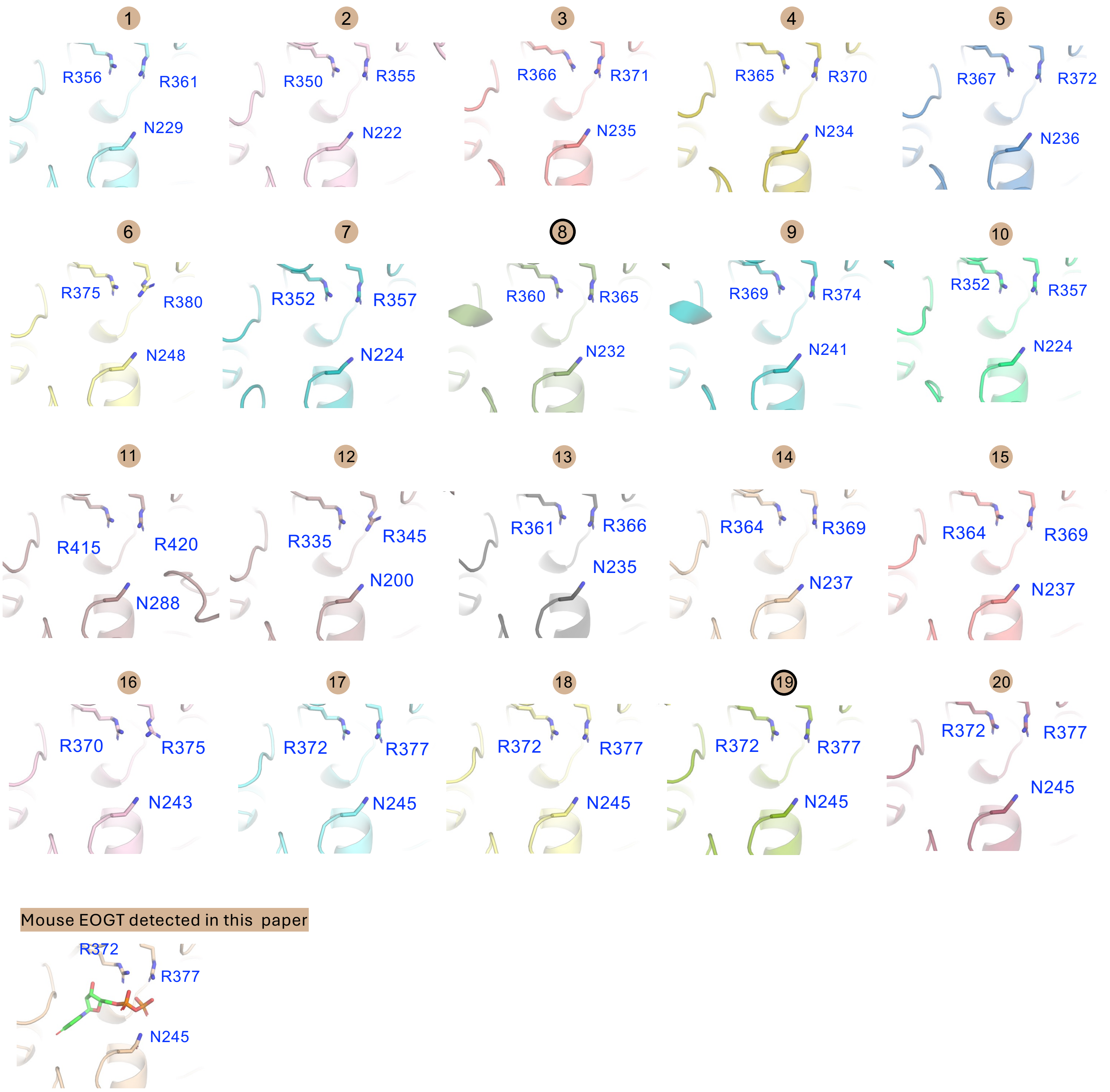

Fig. S6. N–R–R constellations in EOGT and its orthologs identified from the detected homologs.
The structures of EOGT and its orthologs, shown in Figure 4A, were predicted using AlphaFold2. GT61 proteins with experimentally validated enzyme activities are indicated by outlined circles. The corresponding gene and protein lists are presented in Table S2.

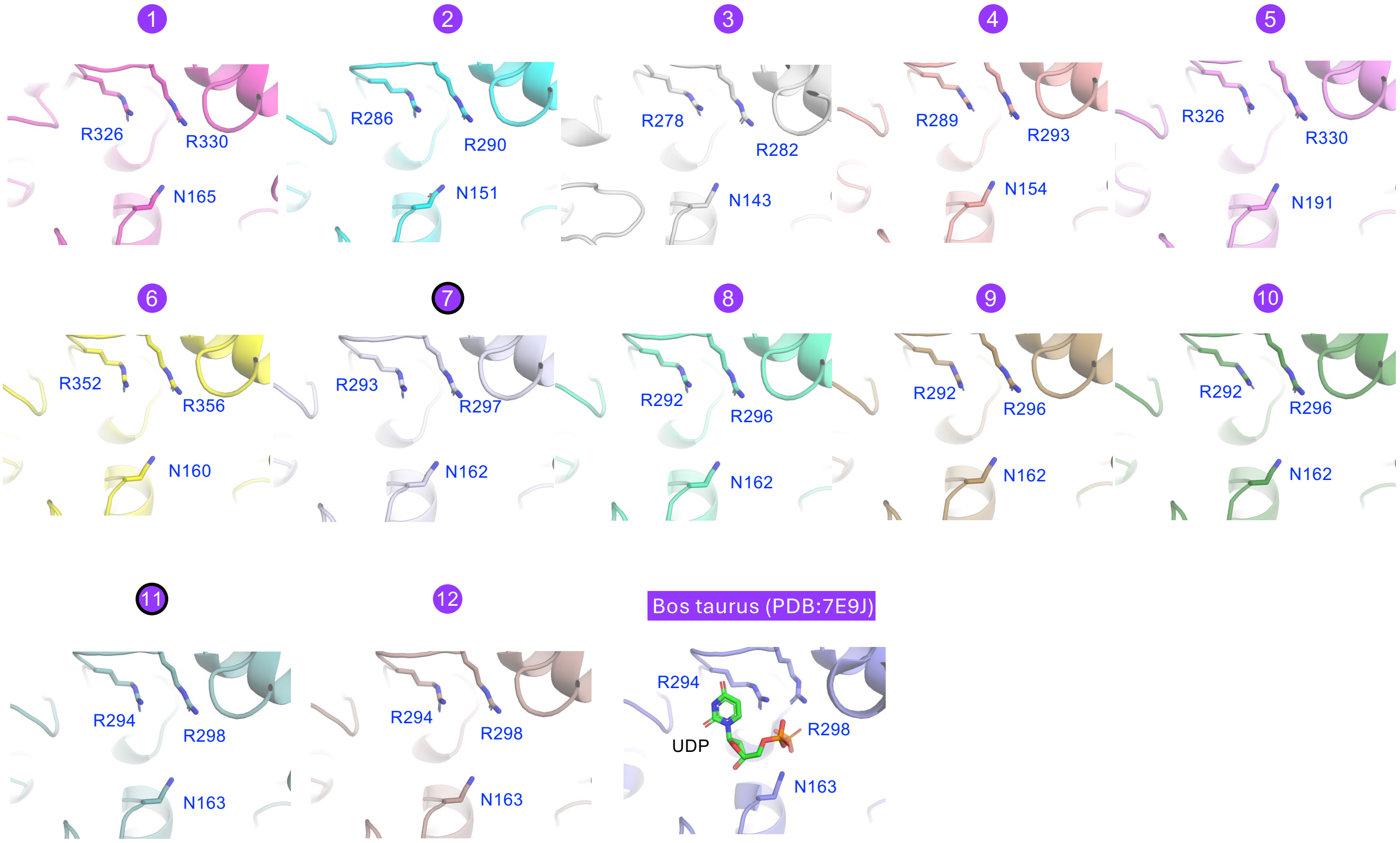

Fig. S7. N–R–R constellations in protein *O*-linked-mannose β1,4-*N*-acetylglucosaminyltransferase 2 (POMGNT2) and its orthologs.
The structures of POMGNT2 and its orthologs, shown in Figure 4A, were predicted using AlphaFold2. GT61 proteins with experimentally validated enzyme activities are indicated by outlined circles. The corresponding gene and protein lists are presented in Table S2.

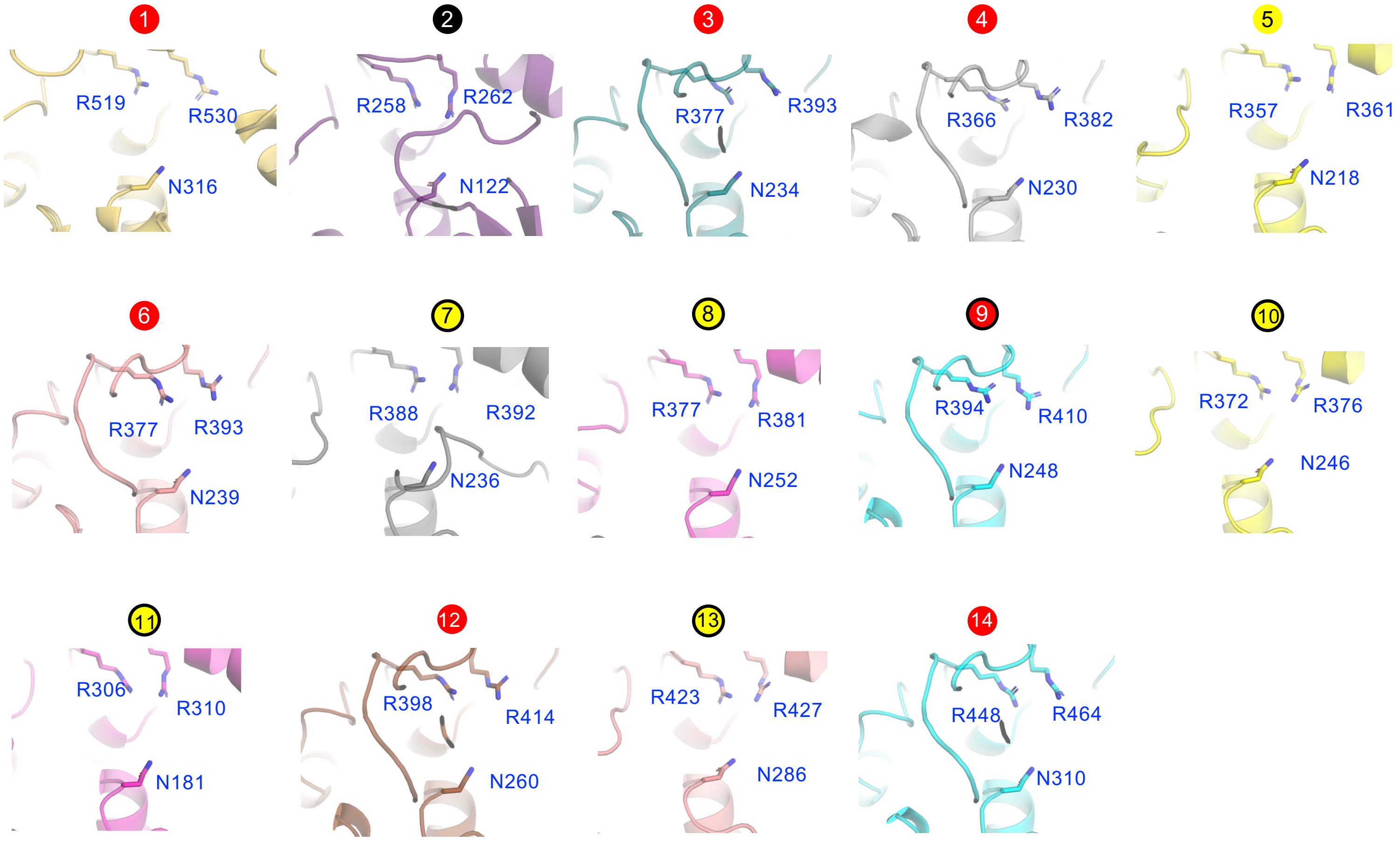

Fig. S8. N–R–R constellations in xylan arabinosyl transferase (XAT)/ xylan 2-*O*-arabinosyltransferase (XAXT)/ xylan β-1,2-*O*-xylosyltransferase (XYXT), core β-1,2-xylosyltransferase (β1,2-XylT), and their orthologs.

The structures of XAT/XAXT/XYXT (yellow circle), core β1,2-XylT (*red* circle), and their orthologs, shown in Figure 4A, were modeled using AlphaFold2. GT61 proteins with experimentally validated enzyme activities are indicated by outlined circles. The corresponding gene and protein lists are presented in Table S2.

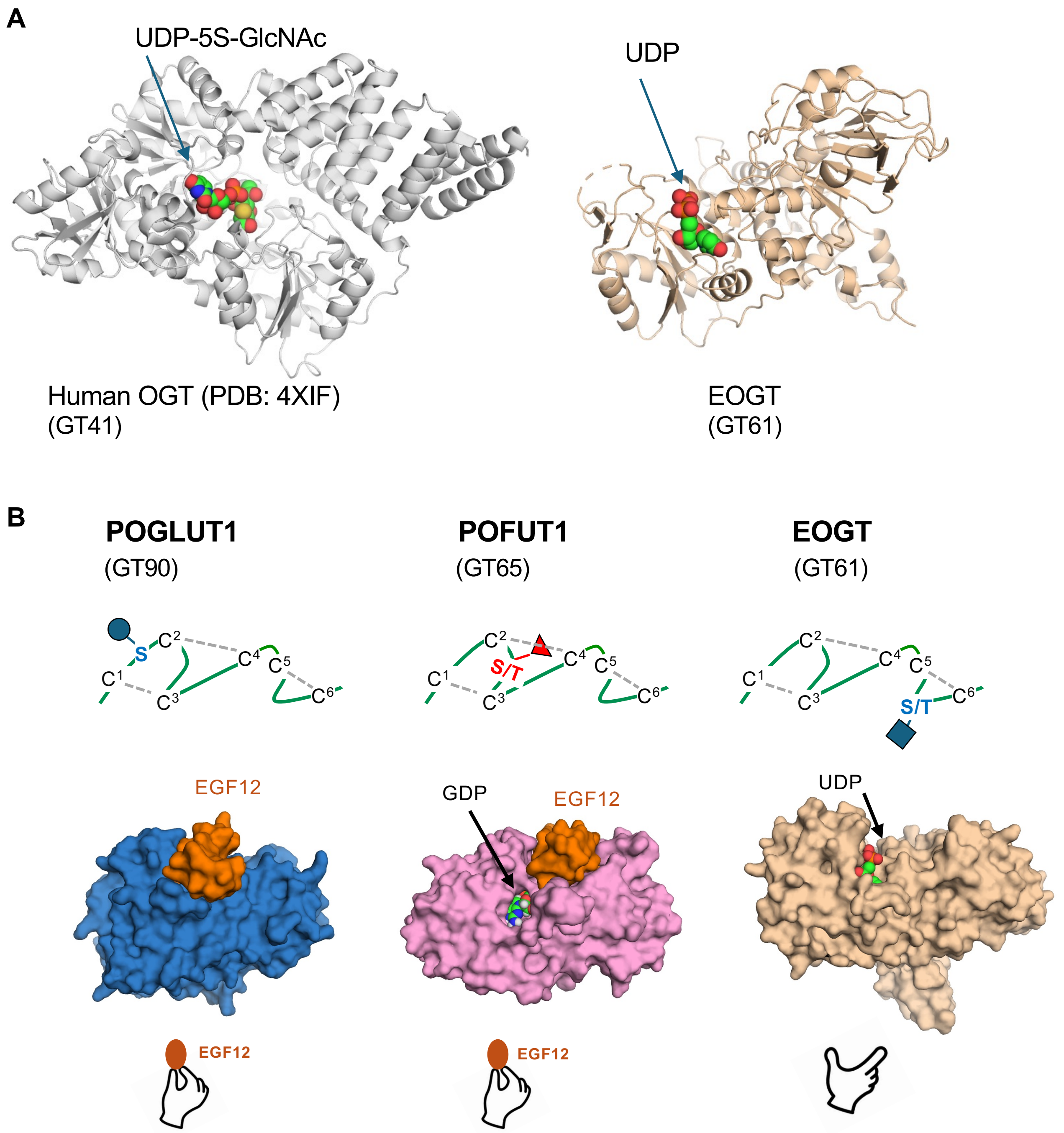

Fig. S9. Differences among GTs related to EOGT functions.
(A) Comparison of *O*-GlcNAc transferases between OGT and EOGT. OGT (*white*; PDB: 4XIF) belongs to the GT41 family, whereas EOGT (*wheat*) belongs to the GT61 family. (B) Comparison of GTs specifically modifying the EGF domain. POGLUT1 (*blue*; PDB: 5L0R) belonging to the GT90 family transfers glucose to serine between C^1 and C^2 in an EGF domain. POFUT1 (*purple*; PDB: 5KY0) belonging to the GT65 family transfers fucose to serine or threonine between C^2 and C^3 in an EGF domain. *Green* line, EGF domain; *gray* dotted line, disulfide bond; C, numbered cysteine in EGF domain; circle (*blue*), glucose; triangle (*red*), fucose; square (*blue*), GlcNAc; oval (*reddish brown*), EGF12.

Supporting Tables

Table S1. Glycosyltransferase (GT61) family shown in Figure 3C.

Enzyme name	Gene	Donor	Reaction	Species		Entry no.
EGF domain-specific O-linked N-acetylglucosamine transferase	Eogt	UDP–GlcNAc	Inverting	Mammal	Mus musculus (mouse)	NP_780522.1
Protein O-linked-mannose beta-1,4-N-acetylglucosaminyltransferase 2	POMGNT2	UDP–GlcNAc	Inverting	Mammal	Bos taurus (bovine)	UniProt: Q5NDF2
Xylan arabinosyl transferase	XAT1	UDP–Arabinose	Inverting	Plant	Triticum aestivum	UniProt: F6IB53
Xylan arabinosyl 2-O-xylosyltransferase 1<br><br>(Xylan 2-O-xylosyl/2-O-arabinosyl transferase)	XAXT1	UDP–Xylose	Inverting	Plant	Oryza sativa (rice)	A0A976SMA0
		UDP–Arabinose	Inverting			
Beta-1,2-xylosyltransferase XYXT1	XYXT1	UDP–Xylose	Inverting	Plant	Oryza sativa subsp. japonica (rice)	UniProt: Q5Z8T8
β-1,2-xylosyltransferase	XYLT	UDP–Xylose	Inverting	Plant	Arabidopsis thaliana (mouse-ear cress)	UniProt: Q9LDH0

Table S2. Gene and protein annotations used in Figure 4.

Enzyme category	No. in Figure	Protein	Gene	Species		Entry no.
EOGT	1	EGF domain-specific O-linked N-acetylglucosamine transferase-like	LOC135342667	Porifera	Halichondria panicea	XP_064395537.1
	2	EGF domain-specific O-linked N-acetylglucosamine transferase-like	LOC136197437	Porifera	Oscarella lobularis	XP_065843270.1
	3	EGF domain-specific O-linked N-acetylglucosamine transferase	LOC5501625	Cnidaria	Nematostella vectensis	XP_048587774.1
	4	EGF domain-specific O-linked N-acetylglucosamine transferase-like	LOC138024706	Cnidaria	Montipora capricornis	XP_068728028.1
	5	EGF domain-specific O-linked N-acetylglucosamine transferase-like	LOC111340563	Cnidaria	Stylophora pistillata	XP_022803155.1
	6	Hypothetical protein RRG08_004947	RRG08_004947	Mollusk	Elysia crispata	GenBank: KAK3769696.1
	7	EGF domain-specific O-linked N-acetylglucosamine transferase	CELE_H12D21.10	Nematoda	Caenorhabditis elegans	UniProt: Q9XTX0
	8	Drosophila melanogaster EGF-domain O-GlcNAc transferase (Eogt), transcript variant A, mRNA	Eogt	Arthropoda	Drosophila melanogaster	NM_134834.3
	9	Hypothetical protein KR054_004230	KR054_004230	Arthropoda	Drosophila jambulina	GenBank: KAH8281953.1
	10	EGF domain-specific O-linked N-acetylglucosamine transferase	HDE_02015	Arthropoda	Halotydeus destructor	GeneBank: KAI1303926.1
	11	EOGT	EOGT, BLAG_LOCUS19228	Cephalochordata	Branchiostoma lanceolatum	UniProt: A0A8K0A074
	12	EGF domain-specific O-linked N-acetylglucosamine transferase	LOC100176976	Urochordata /Tunicata	Ciona intestinalis	XP_002126731.1
	13	Unnamed protein product	KLAPL1V2EN2_LOCUS9303	Cyclostomata /Jawless vertebrates	Lampetra planeri	GenBank: CAM9995323.1
	14	EGF domain specific O-linked N-acetylglucosamine transferase	EOGT	Cyclostomata /Jawless vertebrates	Lethenteron reissneri	XP_061407825.1
	15	EGF domain-specific O-linked N-acetylglucosamine transferase isoform X1	eogt	Vertebrate	Danio rerio	XP_005155877.3
	16	EGF domain-specific O-linked N-acetylglucosamine transferase precursor	eogt	Vertebrate	Xenopus tropicalis	NP_001072691.1
	17	EGF domain-specific O-linked N-acetylglucosamine transferase	EOGT	Vertebrate	Gallus gallus	NP_001026580.1
	18	EGF domain-specific O-linked N-acetylglucosamine transferase isoform X1	EOGT	Vertebrate	Pelodiscus sinensis	XP_075794676.1
	19	EGF domain-specific O-linked N-acetylglucosamine transferase	Eogt	Vertebrate	Mus musculus	NP_780522.1
	20	EGF domain-specific O-linked N-acetylglucosamine transferaseprecursor	Eogt	Vertebrate	Rattus norvegicus	NP_001009502.1
POMGNT2	1	Protein O-linked-mannose beta-1,4-N-acetylglucosaminyltransferase 2-like	LOC136183786	Porifera	Oscarella lobularis	XP_065826612.1
	2	Protein O-linked-mannose beta-1,4-N-acetylglucosaminyltransferase 2-like	LOC138020113	Cnidaria	Montipora capricornis	XP_068723214.1
	3	Protein O-linked-mannose beta-1,4-N-acetylglucosaminyltransferase 2	HDE_04754	Arthropoda	Halotydeus destructor	GenBank: KAI1297162.1
	4	Protein O-linked-mannose beta-1,4-N-acetylglucosaminyltransferase 2-like	LOC118417908	Cephalochordata	Branchiostoma floridae	XP_035679561.1
	5	Protein O-linked-mannose beta-1,4-N-acetylglucosaminyltransferase 2-like isoform X1	LOC136439015	Cephalochordata	Branchiostoma lanceolatum	XP_066290209.1
	6	Protein O-linked-mannose beta-1,4-N-acetylglucosaminyltransferase 2	POMGNT2	Cyclostomata /Jawless vertebrates	Lethenteron reissneri	XP_061421736.1
	7	Glycosyltransferase	ago61	Vertebrate	Danio rerio	GenBank: CAI30873.1
	8	Protein O-linked-mannose beta-1,4-N-acetylglucosaminyltransferase 2	pomgnt2	Vertebrate	Xenopus tropicalis	NP_001086091.1
	9	Protein O-linked-mannose beta-1,4-N-acetylglucosaminyltransferase 2	POMGNT2	Vertebrate	Gallus gallus	NP_001012294.1
	10	Protein O-linked-mannose beta-1,4-N-acetylglucosaminyltransferase 2	POMGNT2	Vertebrate	Pelodiscus sinensis	XP_006125629.2
	11	Protein O-linked-mannose beta-1,4-N-acetylglucosaminyltransferase 2	Pomgnt2	Vertebrate	Mus musculus	NP_001276487.1
	12	Protein O-linked-mannose beta-1,4-N-acetylglucosaminyltransferase 2	Pomgnt2	Vertebrate	Rattus norvegicus	NP_001009437.1
Plant	1	EGF domain-specific O-linked N-acetylglucosamine transferase	C2E21_6145	Chlorophyta	Chlorella sorokiniana	GenBank: PRW45163.1
	2	g12887	g12887	Chlorophyta	Coccomyxa viridis	GenBank: CAL5229540.1
	3	Glycoprotein 2-beta-D-xylosyltransferase	MpGT61.4	Bryophyte	Marchantia polymorpha subsp. Ruderalis	GenBank: BFI29247.1
	4	Unnamed protein product	CSSPTR1EN1_LOCUS8407	Bryophyte	Sphagnum troendelagicum	GenBank: CAK9205972.1
	5	Xylan glycosyltransferase MUCI21-like	LOC131062984	Gymnosperma	Cryptomeria japonica	XP_057852712.2
	6	Beta-1,2-xylosyltransferase RCN11	LOC131073742	Gymnosperma	Cryptomeria japonica	XP_057866228.1
	7	Xylan 2-O-xylosyltransferase 2	XYXT2	Gymnosperma	Pinus taeda (Loblolly pine)	GenBank: UXL82355.1
	8	Glycosyltransferase family 61 protein	GeneID:821344	Angiosperm	Arabidopsis thaliana	NP_001326302.1
	9	Beta-1,2-xylosyltransferase	XYLT	Angiosperm	Arabidopsis thaliana	UniProtKB/Swiss-Prot: Q9LDH0.1
	10	Xylan arabinosyl 2-O-xylosyltransferase 1	XAXT1	Angiosperm	Oryza sativa	A0A976SMA0
	11	Beta-1,2-xylosyltransferase XYXT1	XYXT1	Angiosperm	Oryza sativa subsp. japonica	UniProtKB/Swiss-Prot: Q5Z8T8.1
	12	Beta-1,2-xylosyltransferase RCN11	LOC4345969	Angiosperm	Oryza sativa Japonica Group	NP_001409548.1
	13	Xylan arabinosyl 2-O-xylosyltransferase 1	XAXT1	Angiosperm	Brachypodium distachyon	GenBank: UVC67028.1
	14	Beta-(1,2)-xylosyltransferase	LOC100843487	Angiosperm	Brachypodium distachyon	XP_010235291.2
Proteobacteria	1	Glycosyltransferase family 61 protein	PAE53_18900	Proteobacteria	Sphingobium yanoikuyae	GenBank: WBQ15957.1

Table S3. Pairwise alignment scores between EOGT orthologs and *Mus musculus* EOGT obtained using BLAST2.

Representative sequence	Max Score	Total Score	Query Cover	E value	Per. ident	Acc. Len	Accession
NP_780522.1_Mus musculus	1106	1106	100%	0	100	527	Query_1750633
NP_001009502.1_Rattus norvegicus	1052	1052	100%	0	94.31	527	Query_1750634
XP_075794676.1_Pelodiscus sinensis	866	866	99%	0	76.82	539	Query_1750632
NP_001026580.1_Gallus gallus	862	862	99%	0	74.53	530	Query_1750631
NP_001072691.1_Xenopus tropicalis	789	789	98%	0	69.62	525	Query_1750630
XP_005155877.3_Danio rerio	766	766	95%	0	67.53	519	Query_1750629
XP_061407825.1_Lethenteron reissneri	715	715	93%	0	64.71	519	Query_1750628
CAM9995323.1_Lampetra planeri	706	706	93%	0	64.5	570	Query_1750627
A0A8K0A074 _Branchiostoma lanceolatum	599	599	92%	0	55.12	525	Query_1750625
KAK3769696.1_Elysia crispate	576	576	93%	0	54.95	540	Query_1750623
XP_064395537.1_Halichondria panicea	507	507	86%	1E-179	52.28	518	Query_1750618
XP_065843270.1_Oscarella lobularis	495	495	87%	3E-175	51.37	498	Query_1750619
KAI1303926.1_Halotydeus destructor	478	478	92%	3E-166	48.07	644	Query_1750622
XP_068728028.1_Montipora capricornis	462	462	88%	7E-162	49.79	517	Query_1750616
NM_134834.3_Drosophila melanogaster	452	452	95%	3E-158	46.58	520	Query_1750620
XP_048587774.1_Nematostella vectensis	445	445	89%	3E-155	46.53	516	Query_1750615
KAH8281953.1_Drosophila jambulina	444	444	84%	1E-154	49.67	531	Query_1750621
XP_022803155.1_Stylophora pistillata	442	442	85%	3E-154	48.13	519	Query_1750617
XP_002126731.1_Ciona intestinalis	417	417	83%	2E-144	47.39	507	Query_1750626
Q9XTX0_Caenorhabditis elegans OS=Caenorhabditis elegans OX=6239	385	385	91%	8E-132	43.33	523	Query_1750624

Table S4. Reference list indicated in Figure 4

Enzyme	Species	References
**EOGT**	Drosophila	Muller et al.,2013,PLoS One, 8; Sakaidani et al.,2012,Biochem Biophys Res Commun, 419
	Mouse	Sawaguchi et al.,2017,Elife, 6
**POMGNT2**	Zebrafish	Flannery et al.,2025,bioRxiv,
	Mouse	Nakagawa et al.,2015,Sci Rep, 5; Yagi et al.,2013,Sci Rep, 3
	Human	Yoshida-Moriguchi et al.,2013,Science, 341
**XAT**	Triticum aestivum	Anders et al.,2012,Proc Natl Acad Sci U S A, 109
**XAXT**	Oryza sativa	International Brachypodium,2010,Nature, 463; Zhong et al.,2022,Planta, 256
	Brachypodium distachyon	Zhong et al.,2021,Planta, 254
	Triticum aestivum	Prins & Kosik,2023,Plants-Basel, 12
	Arabidopsis thaliana	Zhong et al.,2025,Plant Cell Physiol,
**XYXT**	Pinus taeda	Zhong et al.,2022,Planta, 256
	Oryza sativa	Zhong et al.,2021,Planta, 254; Zhong et al.,2018,Plant Cell Physiol, 59
	Arabidopsis thaliana	Voiniciuc et al.,2015,Plant Physiol, 169; Zhong et al.,2025,Plant Cell Physiol,
**core β1,2-XylT**	Chlamydomonas reinhardtii	Oltmanns et al.,2019,Front Plant Sci, 10
	Arabidopsis thaliana	Strasser et al.,2000,FEBS Lett, 472

Table S5. Gene and protein annotations used in Figure 5B.

Enzyme name	Gene	Species		Entry no.
Glycosyltransferase	pgtB	Slime mold	Cavenderia fasciculata	UniProt: F4PLZ8
Hypothetical protein DDB_G0290079 [Dictyostelium discoideum AX4]pgtB	pgtB	Slime mold	Dictyostelium discoideum AX4	DDB: 0231870, XP_635901.1
Glycosyltransferase	CYY_002535*	Slime mold	Polysphondylium violaceum	UniProt: A0A8J4Q7U0
Uncharacterized protein	A3770_02p16930*	Chlorophyta	Chloropicon primus	UniProt: A0A5B8MHP4
Tetratricopeptide TPR_2 repeat	C2E21_7179*	Chlorophyta	Chlorella sorokiniana	UniProt: A0A2P6TI43
Gycosyltransferase family 61 protein	PAE53_18900*	Proteobacteria	Sphingobium yanoikuyae	GenBank: WBQ15957.1

*, denotes locus tag.

Table S6. Primers used for site-specific mutagenesis in mouse epidermal growth factor domain-specific *O*-linked *N*-acetylglucosamine transferase (EOGT).

Mutation	Primer sequence	
N245D	Forward	5'-GGTATTGATATGTACCACCACTTCTGT-3'
	Reverse	5'-GTACATATCAATACCTGCATCTAATTT-3'
N245Q	Forward	5'-GGTATTCAGATGTACCACCACTTCTGT-3'
	Reverse	5'-GTACATCTGAATACCTGCATCTAATTT-3'
R372K	Forward	5'-CTTGCAAAAAGCACAGAATACCGGAAA-3'
	Reverse	5'-TGTGCTTTTTGCAAGAATGGTAACTCG-3'
R372L	Forward	5'-CTTGCACTGAGCACAGAATACCGGAAA-3'
	Reverse	5'-TGTGCTCAGTGCAAGAATGGTAACTCG-3'
R375A	Forward	5'-CAGCACAGCATACCGGAAAATCCTGAAC-3'
	Reverse	5'-CGGTATGCTGTGCTGCGTGCAAGAAT-3'
E375Q	Forward	5'-CAGCACACAATACCGGAAAATCCTGAAC-3'
	Reverse	5'-CGGTATTGTGTGCTGCGTGCAAGAAT-3'
Q412E	Forward	5'-TTAGATGAGCTCAGGATCACGCACAAC-3'
	Reverse	5'-CCTGAGCTCATCTAAAAACCCGAGTTC-3'
Q412N	Forward	5'-TTAGATAACCTCAGGATCACGCACAAC-3'
	Reverse	5'-CCTGAGGTTATCTAAAAACCCGAGTTC-3'
H432A	Forward	5'-CTTACCGCCTTACTTTTCCTTCCGGAC-3'
	Reverse	5'-AAGTAAGGCGGTAAGGCCAGCTCCATG-3'
